# Integration of lipidomics and transcriptomics unravels aberrant lipid metabolism and defines cholesteryl oleate as potential biomarker of prostate cancer

**DOI:** 10.1038/srep20984

**Published:** 2016-02-11

**Authors:** Jia Li, Shancheng Ren, Hai-long Piao, Fubo Wang, Peiyuan Yin, Chuanliang Xu, Xin Lu, Guozhu Ye, Yaping Shao, Min Yan, Xinjie Zhao, Yinghao Sun, Guowang Xu

**Affiliations:** 1 Key Laboratory of Separation Science for Analytical Chemistry, Dalian Institute of Chemical Physics, Chinese Academy of Sciences, 116023 Dalian, China; 2 Department of Urology, Shanghai Changhai Hospital, Second Military Medical University, Shanghai, China

## Abstract

In-depth delineation of lipid metabolism in prostate cancer (PCa) is significant to open new insights into prostate tumorigenesis and progression, and provide potential biomarkers with greater accuracy for improved diagnosis. Here, we performed lipidomics and transcriptomics in paired prostate cancer tumor (PCT) and adjacent nontumor (ANT) tissues, followed by external validation of biomarker candidates. We identified major dysregulated pathways involving lipogenesis, lipid uptake and phospholipids remodeling, correlated with widespread lipid accumulation and lipid compositional reprogramming in PCa. Specifically, cholesteryl esters (CEs) were most prominently accumulated in PCa, and significantly associated with cancer progression and metastasis. We showed that overexpressed scavenger receptor class B type I (SR-BI) may contribute to CEs accumulation. In discovery set, CEs robustly differentiated PCa from nontumor (area under curve (AUC) of receiver operating characteristics (ROC), 0.90–0.94). In validation set, CEs potently distinguished PCa and non-malignance (AUC, 0.84–0.91), and discriminated PCa and benign prostatic hyperplasia (BPH) (AUC, 0.90–0.96), superior to serum prostate-specific antigen (PSA) (AUC = 0.83). Cholesteryl oleate showed highest AUCs in distinguishing PCa from non-malignance or BPH (AUC = 0.91 and 0.96). Collectively, our results unravel the major lipid metabolic aberrations in PCa and imply the potential role of CEs, particularly, cholesteryl oleate, as molecular biomarker for PCa detection.

Prostate cancer is one of the most frequently diagnosed malignance in males worldwide, especially in developed countries^1^. It was ranked as the most commonly diagnosed cancer and second leading cause of lethal cancer in American males of year 2014^2^. The early detection of prostate carcinoma suffers from low specificity and sensitivity of PSA, reflected by unnegligible rate of PCa including high-grade PCa among individuals with a PSA level ≤4 ng/ml as well as relatively high rate of non-malignant cases among men with a 4–10 ng/ml PSA level determined by biopsy^3^,^4^. These pitfalls have led to PSA controversy in considering the cost of substantial over-diagnosis and overtreatment following PSA elevation^5^. Therefore it is critical to develop novel diagnostic biomarkers with greater accuracy.

Metabolic reprogramming, including that of lipid metabolism, represents an established signature of cancer biology^6^,^7^. Bioactive lipids and lipid-modified proteins participate in pathogenesis of multiple cancers via lipid signaling networks^8^. Lipidomics approach, which enables a precise characterization of lipid structures and compositions within given cells or organisms, has been widely applied in cancer research^9^. Facilitated by high-throughput lipidomics, the relevance of lipids to cancer pathogenesis in context of, for instance, oncogene MYC overexpression^10^, hypoxia and Ras activation^11^, have been investigated. Meanwhile, the lipid metabolic features associated with breast cancer aggressiveness and progression have been characterized by lipidomics^12^.

In-depth delineation of lipid metabolic atlas in PCa is expected to open new insights into cancer tumorigenesis and progression, and may provide potential biomarker candidates for better diagnosis and prognosis. Existing studies have demonstrated that alterations in lipid metabolic enzymes and pathways, including those of fatty acids^13^,^14^ and cholesterol metabolism^15^,^16^,^17^, are closely associated with PCa. However, comprehensive elucidation of lipid metabolic events and its regulations in PCa remains largely unexplored, especially in context of system-level networks. By far, a panel of lipid metabolites, including (ether-linked) phosphatidylethanolamines, fatty acids, lysophospholipids and other phospholipids, have been proposed as potential PCa biomarkers in distinguishing PCa patients from healthy individuals^18^,^19^,^20^. Nevertheless, most of them failed to correlate with PCa metastasis, aggressivity and benign hyperplasia. Based on metabolic profiling sarcosine has been identified as a potential biomarker to distinguish benign, localized and metastatic PCa^21^. However, the utility of sarcosine remains controversial^22^.

Since the adaptive transformation of lipid metabolism is highly dynamic and involved with complex regulatory networks, a focus on lipids phenotype *per se* remains insufficient. Recently approaches by integrating multi-omics datasets, i.e., information on genome-, proteome-, metabolome-scale etc., have achieved unprecedented insights into complex biological systems. Lipogenic network has been identified associated with hepatocellular carcinoma progression by combined analysis of metabolite and gene expression profiles^23^. By similar approach, the reliance of highly proliferating cancer cells on amino acid glycine has been revealed^24^.

To broaden our understanding of the metabolic alterations of lipid-gene networks in PCa and to identify potential biomarkers, 76 PCa and 19 BPH patients were enrolled in this study (Table 1, supplementary Table S1). Integrated study of lipidomics and transcriptomics (gene and miRNA expression profiling) was performed in paired ANT-PCT tissues from 25 PCa patients (discovery set). The identified biomarker candidates were further externally validated in a cohort including 51 PCa patients and 19 BPH patients (validation set). The workflow of study design is provided in supplementary information (supplementary Fig. S1).

## Results

### Global lipidomics analysis of prostatic tissue

Global lipidomics profiling was applied for qualitative and quantitative characterization of lipidome in 171 prostatic tissue samples acquired from 76 PCa and 19 BPH patients. In total 350 lipid species, spanning 6 cholesteryl ester (CE), 10 ceramide (Cer), 7 diacylglycerol (DAG), 24 free fatty acid (FFA), 9 hexosylceramide (HexCer), 10 lyso-phosphatidylcholine (LPC), 10 lyso-phosphatidylethanolamine (LPE), 3 LPE with alkenyl substituent (LPE-P), 1 lyso-phosphatidylinositol (LPI), 1 lyso-phosphatidylserine (LPS), 1 phosphatidic acid (PA), 37 phosphatidylcholine (PC), 16 PC with alkyl substituent (PC-O), 31 phosphatidylethanolamine (PE), 20 PE with alkenyl substituent (PE-P), 10 phosphatidylglycerol (PG), 15 phosphatidylinositol (PI), 20 phosphatidylserine (PS), 1 PS with alkyl substituent (PS-O), 30 sphingomyelin (SM), 88 triacylglycerol (TAG, including 1 TG-O) were covered. Typical TICs (total ion chromatogram) of lipid profiles of prostatic tissue are provided in supplementary information (supplementary Fig. S2). To enhance in-depth mining of dynamic changes of lipidomic phenotype, radyl substitutes, i.e., acyl/alkyl/alkenyl side-chains were annotated to phospholipid species, and long-chain sphingoid bases as well as N-acyl chains to sphingolipid species. Comprehensive information of all lipids is given in supplementary information (supplementary Table S2).

To ensure the reliability of acquired lipidomics data, quality control (QC) samples were used for data evaluation of both discovery and validation sets. Taking discovery set as an example, approximately 93% lipid species showed a relative standard deviation (RSD) of intensity in QCs (n = 7) below 10%, and more than 99% below 20% (supplementary Fig. S3A). An overview of lipidomic profile in principal component analysis (PCA) score plot (supplementary Fig. S3B) with QCs center-clustered also demonstrates satisfactory robustness and reproducibility of applied method.

### Aberrant lipid metabolic phenotype in PCa

**Figure 1:**
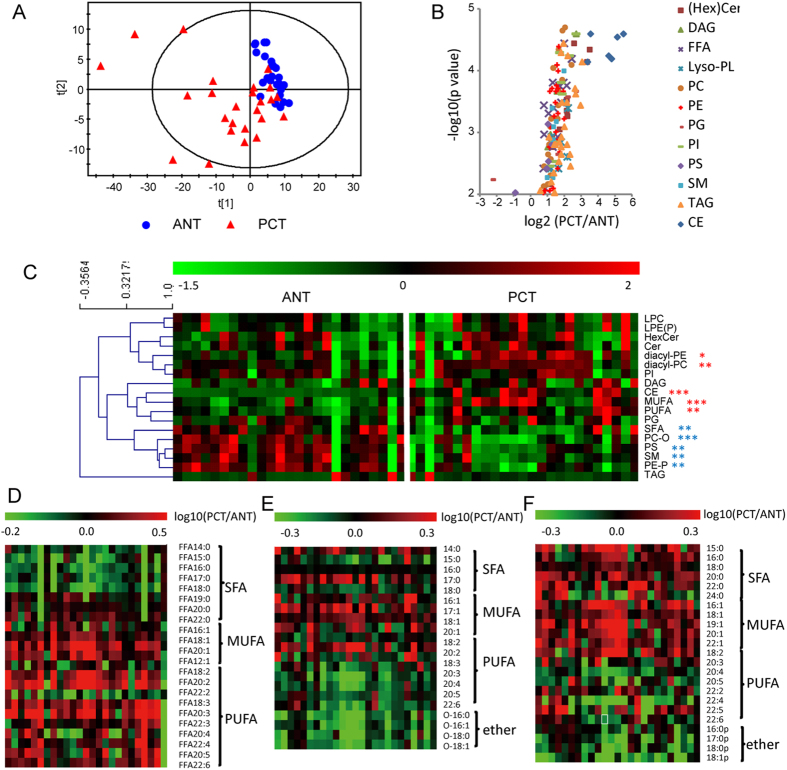
Lipidomics revealed aberrant lipid metabolism in PCa. (**A**) PLS-DA score plot of lipidomic profile of 25 paired ANT- PCT tissue composed of lipid variables after unit variance (uv) scaling pretreatment. (**B**) Volcano plot of 140 significantly altered lipids species (*p* < 0.01). X axis: PCT-to-ANT ratio in log_2_ scale; Y axis: -log_10_ (*p* value); statistical significance was determined by Wilcoxon Signed-Rank test. Cer and HexCer are summarized as (Hex)Cer. (**C**) Heatmap visualization of lipid composition in paired ANT-PCT tissue. Lipid composition was calculated as percentage of amount of (sub)class within entire lipidome and visualized after uv scaling. Each column denotes one prostatic specimen. In total 18 (sub)classes are shown. LPE and LPE-P are summed up as LPE(P). The single LPI, LPS and PA species (i.e., LPI 18:0, LPS 18:0 and PA 36:1) are excluded. Red and blue asterisks indicate significantly increased and decreased, respectively, in PCT vs. ANT. *p < 0.05; **p < 0.01; ***p < 0.001. Statistical significance was evaluated by Wilcoxon Signed-Rank test. (**D**) Heatmap visualization of FA composition in free fatty acid pool. Data shown are paired PCT-to -ANT ratio of FA% within FFA after log_10_ transformation. (**E**,**F**) Heatmap visualization of acyl/alkyl/alkenyl chain composition in PC (**E**) and PE (**F**). Data shown are paired PCT-to-ANT ratio of chain% in PC or PE after log_10_ transformation. In (**D–F**) each column indicates one individual PCa patient. Ether represents alkyl- in PC (**E**) whereas alkenyl-chain in PE (**F**).

To explore the lipidomic profiles in PCa, partial least squares-discriminate analysis (PLS-DA) was performed using lipid abundance data. A pronounced separation between ANT and PCT samples was revealed by PLS-DA score plot (PC = 2; R^2^X = 0.53; R^2^Y = 0.52) (Fig. 1A), indicating distinct alterations of lipid metabolism in PCa. Among 140 differential lipid species (*p* < 0.01), most were dominantly elevated in PCa, as illustrated in volcano plot (Fig. 1B), covering a multitude of lipid classes, including PC, PE, PG, PI, Cer, DAG, CE and FFA, with 1.65–15.87 folds increase in PCT compared to ANT (supplementary Table S3). These observations revealed a prevalent up-regulation of lipid abundance in PCa.

Since lipids constitute vital components in cells, in particular, phospholipids (PLs) as membrane scaffold, changed cell number in tumor proliferation might lead to altered lipid abundance. Therefore we further investigated relative composition changes in PCa lipidome (Fig. 1C, supplementary Table S3). Diacyl-PC and diacyl-PE percentages were significantly increased in PCT whereas ether-linked PCs (alkyl/acyl-PCs, PC-O) and PEs (alkenyl/acyl-PEs, plasmalogens, PE-P) decreased; percentages of free mono- and poly-unsaturated fatty acids (MUFA and PUFA) were elevated, while percentage of free saturated fatty acids (SFA) were reduced. We then investigated distribution of fatty acids (FAs) or radyl residues in lipid pools including FFA and PL categories (PC, PE, PI, PS, PG). In FFA, SFA% was significantly attenuated (PCT/ANT = 0.8, *p* < 0.001) whereas MUFA% and PUFA% were enhanced (PCT/ANT = 1.5 and 1.4, respectively, both *p* < 0.001) (Fig. 1D, supplementary Table S4). In PLs, MUFA-acyl residues were preferably enriched in PCT (PCT/ANT = 1.1–1.4, in 4 PL classes) while PUFA-acyl and ether-linked chains were relatively deficient (PCT/ANT = 0.8–0.9 and 0.6–0.8, respectively, in 4 PL classes) (supplementary Table S4). Detailed fatty chain composition alterations are shown for PC and PE (Fig. 1E,F). This shifted composition hinted that dynamic remodeling might take place in PL metabolism in PCa tumors.

### Network-wide integrated mapping of lipid metabolism in PCa

**Figure 2:**
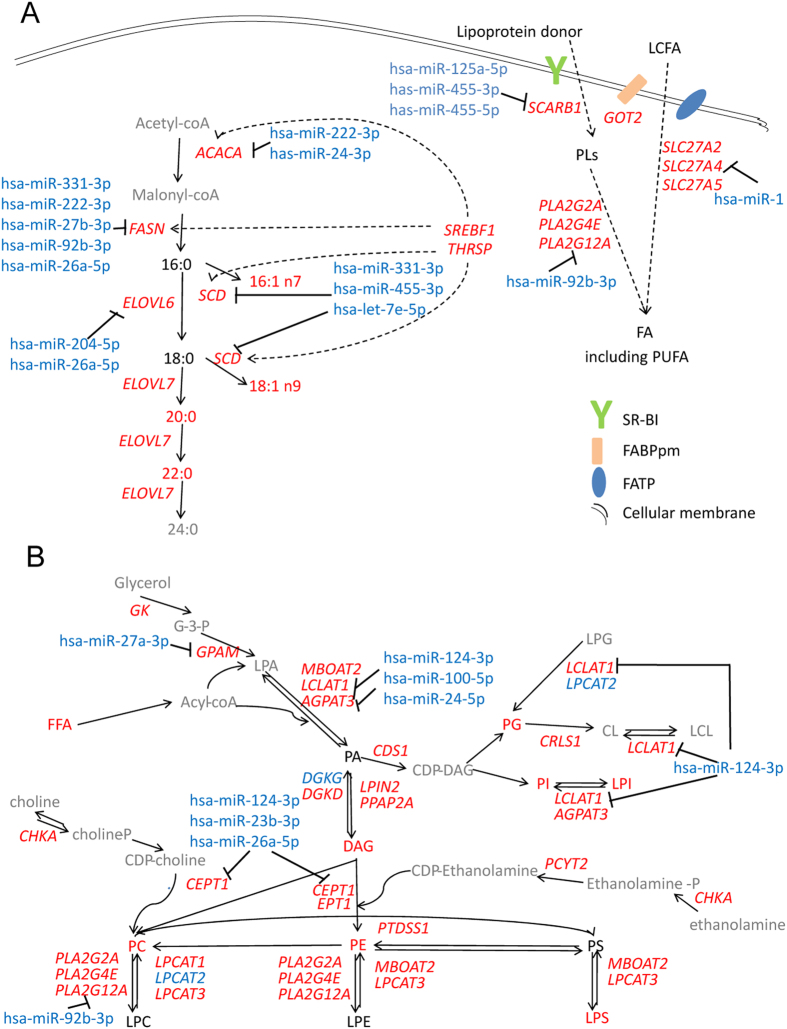
Network-wide integrated pathways depicting fatty acid *de novo *biosynthesis/elongation/desaturation and uptake routes of extracellular PUFA (**A**) as well as phospholipid metabolism (**B**). Red, blue, black and grey colored are significantly increased, significantly decreased, unchanged, and undetected metabolite, or gene, or miRNA, respectively. In italics are gene names. LCFA, long chain fatty acid. Statistical significance was evaluated by Wilcoxon Signed-Rank test.

To uncover potential mechanisms underlying above-described disrupted lipid homeostasis in PCa, we mapped lipid metabolic pathways including lipidome, transcripts alterations and post-transcriptional regulations. 22684 transcripts and 2080 miRNAs were analyzed. Information of lipid related genes and miRNAs is provided in supplementary information Table S5 and Table S6. Since fatty acids assemble a variety of lipid categories, metabolism of free fatty acids was initially investigated. Key genes in *de novo* lipogenesis (DNL), i.e. *ACACA* (acetyl-CoA carboxylase alpha), *FASN* (fatty acid synthase), *SCD* (stearoyl-CoA desaturase) and *ELOVL6*/7 (ELOVL fatty acid elongase 6/7), exhibited significantly increased expression with inverse changes in related miRNAs (Fig. 2A, supplementary Table S5 and Table S6). Transcription factor-encoding genes *SREBF1* (sterol regulatory element binding transcription factor 1, encoding SREBP-1c) and *THRSP* (thyroid hormone responsive) showed significantly elevated expression (Fig. 2A, supplementary Table S5). These data supported up-regulation of DNL activity, a hallmark of cancer pathogenesis, termed “lipogenic phenotype”^25^, which is associated with overall lipid abundance up-regulation in PCa.

In PUFA homeostasis, PUFA biosynthesis from precursor α-linolenic acid and linoleic acid was unaffected in tumor tissues (supplementary Fig. S4). However, expression of *SLC27A2*/*4*/*5* (solute carrier family 27, member 2/4/5), *GOT2* (glutamic-oxaloacetic transaminase 2, mitochondrial) and *SCARB1* (scavenger receptor class B, member 1), encoding fatty-acid-transporter protein (FATP), plasma membrane fatty-acid-binding protein (FABPpm) and SR-BI respectively, were significantly elevated in tumor tissues. Conversely, inhibitory miRNAs showed reduced expression (Fig. 2A, supplementary Table S5 and Table S6). These data suggested PUFA enrichment (Fig. 1C) predominantly relies on extracellular lipid uptake. Since cellular acquirement of dietary PUFA is largely derived from selective PL uptake^26^, we postulated SR-BI to be the key mediator in PUFA accumulation.

We next inspected the metabolic landscape of the PCa lipidome, including phospholipids, ether lipids, sphingolipids, glycerolipids and cholesterol (Fig. 2B, supplementary Fig. S5). The most notable disruption occurred in phospholipid pathway (Fig. 2B). Expression of related genes and miRNAs participating in biosynthesis pathways of PA and downstream PLs, including those of CDP-choline, CDP-ethanolamine, and CDP-DAG pathways, were significantly altered in PCT. Genes including *GPAM* (glycerol 3-phosphate acyltransferase), *MBOAT2* (membrane bound O-acyltransferase domain containing 2), *LCLAT1* (lysocardiolipin acyltransferase 1), *AGPAT3* (1-acylglycerol-3-phosphate O-acyltransferase 3), *CDS1* (CDP-diacylglycerol synthase), *CHKA* (choline kinase alpha), *CEPT1* (choline/ethanolamine phosphotransferase 1), *EPT1* (ethanolaminephosphotransferase 1) and *PCYT2* (phosphate cytidylyltransferase 2) showed significantly increased expression (Fig. 2B, supplementary Table S5). These alterations revealed a strengthened biosynthesis of PLs via the *de novo* pathway (Kennedy pathway)^27^ in PCa (Fig. 2B). Since a reprogrammed composition in membrane phospholipids was observed in PCa (Fig. 1D–F), we further studied alterations of the remodeling pathway (Lands’ cycle)^28^. Phospholipase A2 (PLA2)-related genes, *PLA2G2A*/*4E*/*12A* (phospholipase A2, group IIA /IVE/XIIA), exhibited significantly increased expression, while hsa-miR-92b-3p was reduced. *LPCAT1*/*3* (lysophosphatidylcholine acyltransferase 1/3) and *MBOAT2* (membrane bound O-acyltransferase domain containing 2) expressions were significantly up-regulated whereas *LPCAT2* (lysophosphatidylcholine acyltransferase 2) was down-regulated (Fig. 2B, supplementary Table S5). LPCAT1 is an essential enzyme in dipalmitoyl-PC synthesis; whereas MBOAT2 and LPCAT2 preferentially utilize 18:1-CoA and 20:4-CoA donors, respectively^28^. Since PUFA-acyl chains are predominantly located at the sn-2 position^28^, this activated remodeling characterized by coordinated actions of PLA2s and lysophospholipid acyltransferase (LPLATs) (i.e. enhanced hydrolysis by PLA2s and selective reacylation by LPLATs), contributed to increased MUFA% and reduced PUFA% in PLs in PCa (Fig. 1E,F and supplementary Table S4). Liberated PUFAs from PLs served as the direct source of enriched free PUFA (Fig. 2A). This remodeling pattern was also observed in ether lipid pathway (supplementary Fig. S5) as revealed by alterations of a same set of genes expressions in relation to PLA2s and LPLATs, and was in accordance with reduction of ether-linked chain% in PLs (Fig. 1E,F and supplementary Table S4).

### Most prominent CEs accumulation in PCa and its association with cancer progression

**Figure 3:**
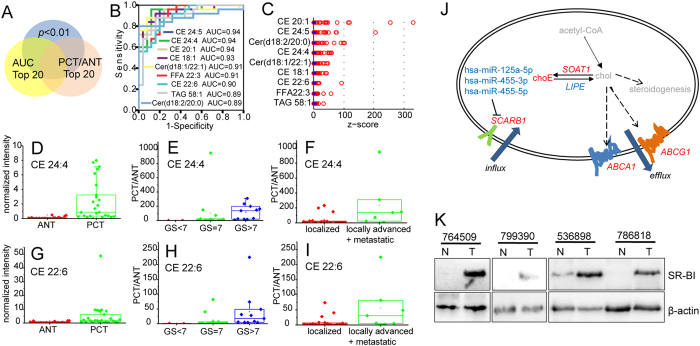
CEs strikingly accumulated in PCa tumor tissues and significantly associated with prostate malignity progression. (**A**) Scheme of selecting most prominently elevated lipid species in PCa. (**B**) ROC curves of selected 9 lipids for differential diagnosis of PCT vs. ANT. (**C**) Z-score plot of selected 9 lipids. Z-value was calculated as (*X*-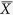)/SD after uv scaling. Blue dot and red circle represent ANT and PCT respectively. **(D**,**G)** Box plot of normalized intensity of CE 24:4 (**D**) CE 22:6 (**G**) in paired ANT and PCT. Intensity of CE species was normalized to internal standard (IS) TAG (15:0/15:0/15:0). **(E**,**H)** Box plot of PCT-to-ANT ratio of CE 24:4 (**E**), CE 22:6 (**H**) in Gleason Score (GS) stages, i.e., GS < 7, GS = 7 and GS > 7. (**F**,**I**) Box plot of PCT-to-ANT ratio of CE 24:4 (**F**), CE 22:6 (**I**) in metastatic grades, i.e., localized, locally advanced + metastatic. (**J**) Integrated pathway depicting dysregulated cholesterol homeostasis in PCa. Red, blue and grey colored are significantly increased, significantly decreased and undetected metabolite, or gene, or miRNA, respectively. In italics are gene names. Statistical significance was evaluated by Wilcoxon Signed-Rank test. (**K**) Immunoblot of endogenous SR-BI in four paired PCT (T) and ANT (N) samples. β-actin was used as a loading control.

On the basis of the landscape that lipids were prevalently up-regulated in PCa, we further sought to determine the most elevated lipids in PCa and to identify potential lipid biomarker candidates according to scheme shown in Fig. 3A. Collectively 9 lipids were selected which simultaneously met the following criterions, i.e., (i) *p* value < 0.01; (ii) top 20 AUC of ROC curve; and (iii) top 20 of PCT/ANT folds change in lipid abundance. They were 5 CEs, 2 Cers, 1 FFA and 1 TAG (supplementary Table S7). ROC curves (Fig. 3B) and Z-score plot (Fig. 3C) of these lipids were generated and converged upon CEs. AUCs of the 5 CE species were 0.90–0.94, showing high discriminant power in distinguishing PCa from non-tumor (Fig. 3B, supplementary Table S7). CEs also exhibited greatest expression increases (10.5–45.5 folds) in PCT compared to ANT (supplementary Table S7). These results suggest that CEs may serve as potential biomarker candidates for PCa diagnosis.

We then explored links between lipid metabolites and PCa progression. Histopathological paradigms regarding to Gleason Score (GS) grades and metastatic grades, i.e., localized, locally advanced and metastatic grade, were investigated for potential lipidomic linkage. Among differentially expressed lipids, 9 lipid species (2 CEs, 6 PUFAs and 1 PG) significantly correlated with GS grades (supplementary Table S8a) whereas 10 species (3 CEs and 7 TAGs) significantly correlated with metastatic grades (supplementary Table S8b). Accumulation of CE 24:4 and CE 22:6 were significantly correlated with both GS and metastatic grades and exhibited most potent increases during PCa progression (Fig. 3D–I, supplementary Table S8). CE 24:4 level showed 48.5 folds increase in GS > 7 tumors and 149.9 folds increase in locally advanced as well as metastatic tumors compared to paired ANT (Fig. 3E,F, supplementary Table S8). The increase of CE 22:6 level were 23.6 and 46.7 folds, respectively (Fig. 3H,I, supplementary Table S8). These findings further supported CEs as potential biomarker candidates for PCa diagnosis and disease progression.

### CEs accumulation may be attributed to SR-BI mediated CEs uptake

These findings led us to investigate the mechanisms of disruption in cholesterol homeostasis (Fig. 3J), which is modulated by processes including cholesterol biosynthesis, esterification, catabolism (steroidogenesis), influx, and efflux^16^,^29^. Gene expression of *HMGCR* (HMG-CoA reductase), the major committed gene in cholesterol synthesis (mevalonate pathway) was not changed (supplementary Fig. S5). In interconversion of free and esterified forms of cholesterol, *SOAT1* (sterol O-acyltransferase 1) expression significantly increased whereas *LIPE* (lipase, hormone-sensitive) decreased (Fig. 3J). There was significantly increased gene expression of the ATP-binding cassette transporters, *ABCG1* (ATP-binding cassette, sub-family G, member 1) and *ABCA1* (ATP-binding cassette, sub-family A, member 1), which are involved in lipid efflux^30^,^31^. In cholesterol influx, *SCARB1* and related miRNAs^32^ including has-miR-125a-5p/455-3p/455-5p were significantly increased and decreased, respectively (Fig. 3J). *SCARB1* encodes SR-BI, which is a key transporter in selective CEs uptake from high-density lipoproteins (HDLs) and is highly expressed in steroidogenic tissues^33^. These data suggested that, elevated esterification, attenuated lipolysis and enhanced influx via SR-BI might contribute to aberrant CEs accumulation. In particular, increased *SCARB1* is highly correlated with CEs accumulation (91.7% of overall prediction accuracy, evaluated by binary logistic regression analysis). Immunoblot showed that SR-BI was overexpressed in multiple PCa primary tumor tissues, compared to matched normal adjacent tissues (Fig. 3K). These data suggested SR-BI to be a key player in aberrant deposition of intratumoral CEs.

### Validation of CEs as PCa biomarkers

**Figure 4:**
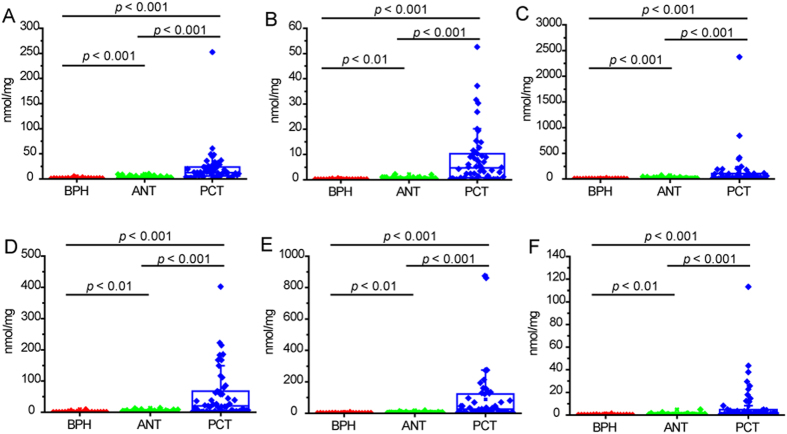
Box plot of metabolite level of 6 CE species in BPH, paired ANT and PCT tissues in validation set. CE was quantified by normalization to CE 13:0. (**A**) CE 18:1; (**B**) CE 20:1; (**C**) CE 22:6; (**D**) CE 24:4; (**E**) CE 24:5; (**F**) CE 28:5. Statistical significance was determined by Wilcoxon Signed-Rank test (PCT vs. ANT) or Mann-Whitney test (BPH vs. ANT, BPH vs. PCT).

Finally, we validated CEs accumulation in an external validation cohort. CEs remarkably accumulated in PCT versus ANT and BPH tissues (Fig. 4). Six CE species showed 7.2–28.8 folds increase in PCT compared to paired ANT (all *p* values < 0.001), and 17.0–76.3 folds increase in PCT compared to BPH (all *p* values < 0.001). CEs robustly distinguished PCa from the non-malignant group (BPH and ANT) (AUCs = 0.84–0.91) (Table 2). The utility of CEs to discriminate PCT from BPH was weighed against that of PSA. CEs exhibited AUCs of 0.9–0.96, superior to PSA (AUC = 0.83) with greater specificity (0.81–1.0 vs. 0.69) (Table 2). Particularly, cholesteryl oleate (CE 18:1) showed highest AUCs in discriminant classification of PCT vs. non-malignance (AUC = 0.91, sensitivity = 0.78, specificity = 0.90) and PCT vs. BPH (0.96, 0.90, 0.94) (Table 2). In addition, authentic standard of stable isotope labeled cholesteryl oleate is commercially available, which is a prerequisite for accurate absolute quantification and is required in large-scaled validation. Therefore, cholesteryl oleate represents the most promising marker and may serve as a complement to PSA in PCa detection. Our findings from both the discovery and validation cohorts indicate CEs may be potential biomarkers for PCa diagnosis, with the most prominent potential of cholesteryl oleate.

## Discussion

Systematic elucidation of lipid metabolism and its underlying regulatory mechanism in tumorigenesis and progression is of critical significance. In this study, We investigated lipid metabolic alterations in PCa by integration of lipidomics and transcriptomics. Direct comparison of adjacent nontumor and tumor tissue counterparts enabled diminished potential effect derived from individual difference, thereby to facilitate the identification of etipathologically-relevant differential molecules. The availability of strictly enrolled tissue samples in prostatic specimen biobank allowed simultaneous profiling of lipidome, transcriptome and histopathological evaluation of same specimen. Although a large body of studies has been reported concerning lipid metabolism in PCa, they are predominantly performed on the genetic level and limited to a few lipid metabolites. To the best of our knowledge, this is the first report of network-wide mapping of lipid metabolism in PCa by integrating lipidomics and transcriptomics data.

Firstly, a prevalence of abundance up-regulation was depicted in PCa lipidome (Fig. 1B), which was associated with exacerbated DNL (Fig. 2A) and phospholipids biosynthesis Kennedy pathway (Fig. 2B). “Lipogenic phenotype”^25^ is universally present in neoplastic transformation or progression of many human malignances and may be regulated by SREBP-1c and AMPK^34^. Fueled by activated DNL, biosynthesis of structural phospholipids for membrane architectures was enhanced in PCa (Fig. 2B). Besides provision of FA precursors for membrane biogenesis and energy storage required in highly proliferative tumor cells, neoplastic lipogenesis *per se* possess oncogenic nature in tumor growth and survival^25^.

Secondly, reprogrammed composition patterns were identified in FFA pool and membrane phospholipids (Fig. 1D–F), which was closely related to the activated PL remodeling (Fig. 2B). PL remodeling affects membrane fluidity and may be implicated in cancer cell signaling via caveolin or lipid rafts, and may affect chemotherapy outcomes^35^. The proportion of sn-2 arachidonoyl (20:4) containing PC in membrane component was reported inversely related to Akt (protein kinase B) activity by suppressing its phosphorylation^36^. Thus decreased PUFA% in PLs detected in our study (Fig. 1E,F and supplementary Table S4) might also impact the proliferation and survival of PCa tumor cells via Akt pathway. The major player in PL remodeling, PLA2s, participate in tumor proliferation, invasion, metastasis and angiogenesis through cascade signaling^37^. The sPLA2 (secretory PLA2) overexpression in androgen-independent PCa is previously reported^38^. Free PUFA was found enriched in both terms of abundance and its proportion (Fig. 1C), which may be largely from increased influx of lipoprotein PLs via SR-BI followed by enhanced PLA2s-mediated hydrolysis (Fig. 2A). In arachidonic acid (AA) metabolism, genes *ALOX15B* (arachidonate 15-lipoxygenase, type B) and *PTGES3* (prostaglandin E synthase 3), involved in COX (cyclooxygenase) and LOX (lipoxygenase) pathways showed significantly increased expression in PCa (supplementary Fig. S4), probably suggesting an enhanced synthesis of downstream bioactive metabolites. Interestingly, PUFA showed a high correlation with PCa aggressiveness (supplementary Table S8a). It may be attributed to pro-inflammatory eicosanoids derived from AA metabolism (prostaglandins and leukotrienes), which mediate cross-talk between neoplastic transformed cells and surrounding cells^39^.

Finally and also most importantly, CEs were found most prominently accumulated in PCT, and moreover, indicative of PCa progression. It was closely associated with dysregulated cholesterol homeostasis, in particular, SR-BI mediated CEs uptake. As a cholesterol reservoir, CEs accumulation avoids potential cytotoxicity of excess free cholesterol^29^ and may participate in intracrine steroidogenesis in castration-resistant PCa (CRPC)^40^. Elevated CEs may reflect enhanced lipid droplet biogenesis, which is implicated in inflammation and neoplastic transformation^41^. It was previously reported that CEs accumulation in PCa may be a consequence of PTEN loss and PI3K/AKT/mTOR pathway activation, further regulating SREBP and increased cholesterol uptake via low density lipoprotein receptor (LDLr)^17^. However, in our study, CEs accumulated in PCa biopsies without PTEN loss. Though PTEN loss is common in western PCa populations, it is infrequent in Chinese patients^42^. Therefore, alternative mechanisms circumventing PTEN loss but resulting in CEs accumulation may underlie PCa pathogenesis. Based on our findings and previous work implicating the role of SR-BI in PCa signaling^43^, SR-BI may represent a rational therapeutic target in PCa treatment.

Identification of novel molecular biomarkers for improved cancer diagnosis and prognosis, especially for effective discrimination of benign and malignant tumor, is significant for clinical research. In this study, CE species effectively distinguished PCa from non-tumor in discovery set with 0.90–0.94 AUCs (Fig. 3B). It was further confirmed in external validation set with 0.84–0.91 AUCs (Table 2). Further, compared to PSA, CEs showed better discriminant capacity in classification of benign and malignant disease with 0.9–0.96 AUCs (Table 2). Specifically, cholesteryl oleate was highlighted as the most promising molecular marker with highest AUCs in distinguishing PCT from non-malignance or BPH (AUC = 0.91 and 0.96, respectively). The identification of potential diagnostic role of cholesteryl oleate may improve PCa detection in clinical setting.

In summary, by integrated study of lipidomics and transcriptomics (gene and miRNA expression), we have identified aberrant lipid metabolism in PCa carcinogenesis and progression, including widespread up-regulation of lipid abundance and reprogramming of lipid composition in distinct lipid pools, driven by activated lipogenesis, lipid uptake and phospholipids remodeling. Specifically, CEs were found most prominently accumulated in PCa and significantly associated with cancer progression, which was driven by enhanced CEs uptake via SR-BI. Further more, CEs were underscored as potential diagnostic biomarkers of PCa in both discovery and validation sets. In particular, cholestryl oleate was defined as the most promising marker with highest discriminant capacity and could be used as a complement to PSA. This finding may lead to improved PCa diagnosis and prognosis. Further studies are warranted to confirm CEs accumulation in large-scaled cohorts with special focus on cholesteryl oleate, and to understand the precise regulatory mechanisms governing CEs accumulation. Besides, tissue samples are less-obtainable than biofluids, further studies are needed to know potential of the biofluid CEs as potential biomarkers. Additionally, development of non-invasive detection techniques of CEs such as imaging technique would be a more useful way for PCa diagnosis.

## Methods

### Chemicals

Liquid chromatography or mass spectrometry grade acetonitrile, isopropanol, methanol (MeOH), and chloroform (CHCl_3_) were purchased from Merck (Darnstadt, Germany). Ammonium acetate and methyl tert-butyl ether were purchased from Sigma-Aldrich (St. Louis, MO, USA). Ultrapure water was prepared by Milli-Q system (Millipore; Billerica, MA, USA). Lipid internal standards (IS) of phosphatidylcholine (PC) (19:0/19:0), phosphatidylethanolamine (PE) (17:0/17:0), lyso-phosphatidylcholine (LPC) 19:0, sphingomyelin (SM) (d18:1/12:0), triacylglycerol (TAG) (15:0/15:0/15:0), ceramide (Cer) (d18:1/17:0), free fatty acid (FFA) 16:0-d3, FFA 18:0-d3 and CE 13:0 were purchased from Avanti (Alabaster, AL, USA) or Sigma. Stock solutions of all standards were prepared in solvent CHCl_3_/MeOH = 2:1 and stored in −20 °C before use.

### Clinical prostatic specimens

All participants were treatment-naïve and enrolled from Shanghai Changhai Hospital (Shanghai, China) with written informed consent. The protocol of the study was approved by Institutional Review Board of the Shanghai Changhai Hospital, Second Military Medical University, Shanghai, China. All experiments were conducted in accordance with the approved guidelines. All paired ANT and PCT tissues were obtained by radical prostatectomy. Among BPH tissues, nine were collected by radical prostatectomy from urinary bladder carcinoma patients with concomitant BPH while the remaining ten were obtained from BPH patients by laser surgery. Examination of H&E (hematoxylin and eosin stained) slides was performed for clinical-histological diagnosis. Sample quality was ensured by examination of H&E slides for high-density foci of tumor cells and absence of contamination from histopathologically normal tissue. Detailed information of all participants are provided in supplemental data (supplementary Table S1). All prostatic tissues were stored in −80 °C until analysis.

### Non-targeted lipidomics

Non-targeted LC-MS based lipidomics analysis of prostatic tissues was performed as previously described with minor modifications^44^. Briefly, frozen prostatic tissues (8.5 ± 2.0 mg in discovery set, 9.4 ± 1.3 mg in validation set) were first spiked with IS solution (in MeOH) containing 8 lipid standards: LPC 19:0, SM (d18:1/12:0), Cer (d18:1/17:0), PE (17:0/17:0), PC (19:0/19:0), TAG (15:0/15:0/15:0), FFA 16:0-d3 and FFA 18:0-d3. The mixture was homogenized using ball mill (mixer mill MM400; Restch; Haan, Germany) followed by addition of methyl tert-butyl ether and one-hour shaking. After phase breaking using water, the up-layer was collected and freeze-dried. Samples were dissolved and spiked with CE 13:0 (in validation set) prior to analysis. Quality control (QC) samples were prepared by pooling equal amounts of lipid extracts from every sample, divided into aliquots.

A hyphenated liquid chromatography-mass spectrometry (LC-MS) system equipped with ACQUITY^TM^ ultra-performance liquid chromatography (UPLC) (Waters; Milford, MA, USA) and AB Sciex tripleTOF 5600 plus mass spectrometer (AB Sciex; Framingham, MA, USA) was employed for global lipidomics profiling. QC samples were analyzed every 8 samples within run.

Lipid identities were determined based on accurate mass, chromatographic retention and tandem mass spectrometry (MS/MS) fragmentation patterns, facilitated by software Lipidview^TM^ (Version 1.2, AB Sciex). Nomenclature and abbreviations of lipid species followed the classification system proposed by LIPIDMAPS^45^.

### Transcriptomics analysis

Transcriptomics analysis was performed by RNA-seq as previously described^46^.

### Immunoblotting

Immunoblot analyses were performed utilizing standard methods. Briefly, cells were lysed in RIPA (radioimmunoprecipitation assay) buffer containing phosphatase inhibitors (Sigma). Proteins were separated by SDS-PAGE (sodium dodecyl sulphate-polyacrylamide gel electrophoresis) and transferred onto a polyvinylidene fluoride (PVDF) membrane (Bio-Rad; Hercules, CA, USA). Membranes were probed with specific primary antibodies and then with peroxidase-conjugated secondary antibodies. Bands were visualized by chemiluminescence (Thermo Scientific; Waltham, MA. USA). Antibodies used in this study were against SR-BI (1:300, Origene; TA507135S; Rockville, MD, USA), and β-actin (1:2000, Cell Signaling Technology, 4967; Beverly, MA, USA).

### Data processing and Statistics

To describe lipid abundance, acquired signal intensity (peak area) of all lipid species were normalized to corresponding IS and tissue weights for relative quantification. Specifically, CE was quantified by normalization to IS CE 13:0 in validation set. To ensure the reliability of acquired lipidomics data, QC samples (n = 7) were used for evaluation of data quality. All lipid variables with a RSD < 20% in QCs were used for subsequent analysis. Lipid composition was calculated as percentage of a defined lipid (sub)class within entire lipidome. Fatty chain composition of phospholipid categories was calculated based on identified radyl substitutes, i.e., acyl/alkyl/alkenyl-side chains in lipid species. SIMCA-P 11.5 (Umetrics; Umeå, Sweden) was employed for multi-variable analysis, including principal component analysis (PCA) and partial least squares-discriminate analysis (PLS-DA) with unit variance (uv) scaling. Heatmap visualization and statistics including Kruskal-Wallis test and Mann-Whitney test were conducted by the open-source software MultiExperiment Viewer (MeV, version 4.9.0)^47^. Wilcoxon Signed-Rank test and Z-score plot generation were performed by MATLAB (MathWorks; Natick, MA, USA). A *p*-value < 0.05 was set as statistically significant. Binary logistic regression analysis, Pearson correlation analysis and generation of ROC curves were performed by SPSS 13.0 for windows (IBM, Bethesda, MD, USA). Database resources KEGG^48^ and miRTarBase^49^ as well as published references were referred to for systematic integration of metabolic pathway networks containing lipid metabolites, genes and miRNAs.

## Additional Information

**How to cite this article**: Li, J. *et al.* Integration of lipidomics and transcriptomics unravels aberrant lipid metabolism and defines cholesteryl oleate as potential biomarker of prostate cancer. *Sci. Rep.* **6**, 20984; doi: 10.1038/srep20984 (2016).

## Supplementary Material

Supplementary Information

## Figures and Tables

**Table 1:**
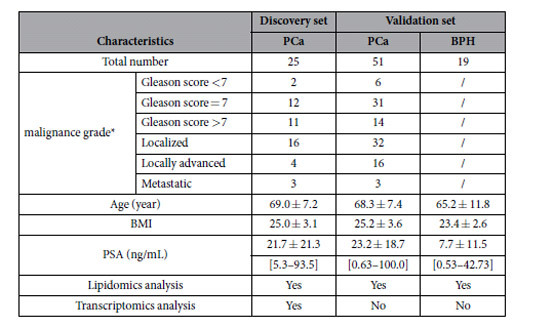
Clinical characteristics and applied analysis of patients enrolled in the study.

^*^malignance information concerning metastatic grades of two samples in discovery set was missing.

**Table 2:** 

discriminant diagnosis	Variable	AUC	Std. Error	95% Confidence Interval		Sensitivity	Specificity
				Lower	Upper		
PCT vs. (BPH + ANT)	CE 18:1	0.91	0.03	0.86	0.97	0.78	0.90
	CE 20:1	0.89	0.03	0.83	0.96	0.73	0.96
	CE 22:6	0.86	0.03	0.79	0.92	0.73	0.81
	CE 24:4	0.88	0.03	0.82	0.95	0.67	0.97
	CE 24:5	0.88	0.03	0.81	0.94	0.71	0.94
	CE 28:5	0.84	0.04	0.76	0.91	0.67	0.91
PCT vs. ANT	CE18:1	0.90	0.03	0.83	0.96	0.75	0.90
	CE20:1	0.88	0.04	0.81	0.95	0.73	0.94
	CE22:6	0.82	0.04	0.74	0.90	0.61	0.90
	CE24:4	0.87	0.04	0.79	0.94	0.67	0.96
	CE24:5	0.86	0.04	0.78	0.93	0.71	0.92
	CE28:5	0.81	0.04	0.73	0.90	0.67	0.90
PCT vs. BPH	PSA	0.83	0.07	0.70	0.97	0.94	0.69
	CE18:1	0.96	0.02	0.91	1.00	0.90	0.94
	CE20:1	0.94	0.03	0.88	0.99	0.80	1.00
	CE22:6	0.95	0.03	0.90	1.00	0.88	0.94
	CE24:4	0.93	0.03	0.86	0.99	0.90	0.88
	CE24:5	0.93	0.03	0.87	0.99	0.86	0.94
	CE28:5	0.90	0.04	0.82	0.98	0.88	0.81

Statistical information of ROC analysis in validation set for discriminant diagnosis of PCT vs. combined BPH and ANT tissues, as well as PCT vs. ANT using CE species; and for discriminant diagnosis of PCT vs. BPH tissues using CE species and PSA.
