# Supplementary material for: Integration of lipidomics and transcriptomics unravels aberrant lipid metabolism and defines cholesteryl oleate as potential biomarker of prostate cancer
Source: Sci Rep. 2016 Feb 11;6:20984. doi: 10.1038/srep20984 (PMC4750101; doi:10.1038/srep20984)
Supplement: Supplementary Information [file srep20984-s1.pdf]

SUPPLEMENTARY INFORMATION

Integration of lipidomics and transcriptomics unravels aberrant lipid metabolism and defines cholesteryl oleate as potential biomarker of prostate cancer

Jia Li[a,1], Shancheng Ren[b,1], Hai-long Piao[a], Fubo Wang[b], Peiyuan Yin[a], Chuanliang Xu[b], Xin Lu[a], Guozhu Ye[a], Yaping Shao[a], Min Yan[a], Xinjie Zhao[a], Yinghao Sun[b,2], Guowang Xu[a,2]

[a] Key Laboratory of Separation Science for Analytical Chemistry, Dalian Institute of Chemical Physics, Chinese Academy of Sciences, 116023 Dalian, China.

[b] Department of Urology, Shanghai Changhai Hospital, Second Military Medical University, Shanghai, China

[1] contributed equally to this paper.

[2] To whom correspondence should be addressed.

Guowang Xu: Tel/Fax: +86-411-84379559. xugw@dicp.ac.cn.

Yinghao Sun: sunyh@medmail.com.cn

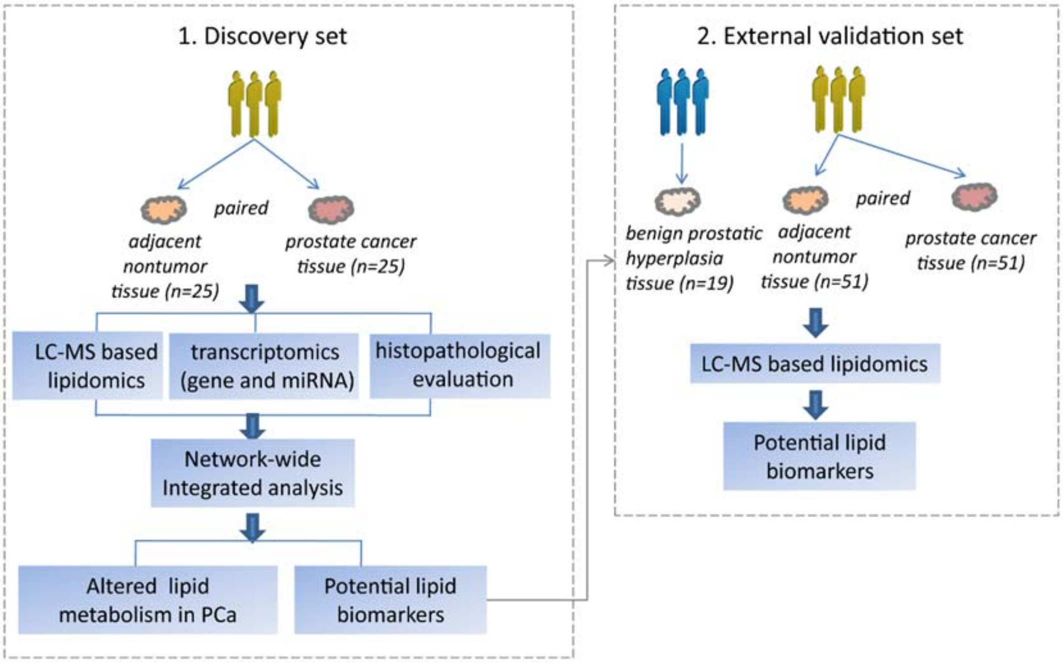

Fig. S1. Workflow diagram of study design.

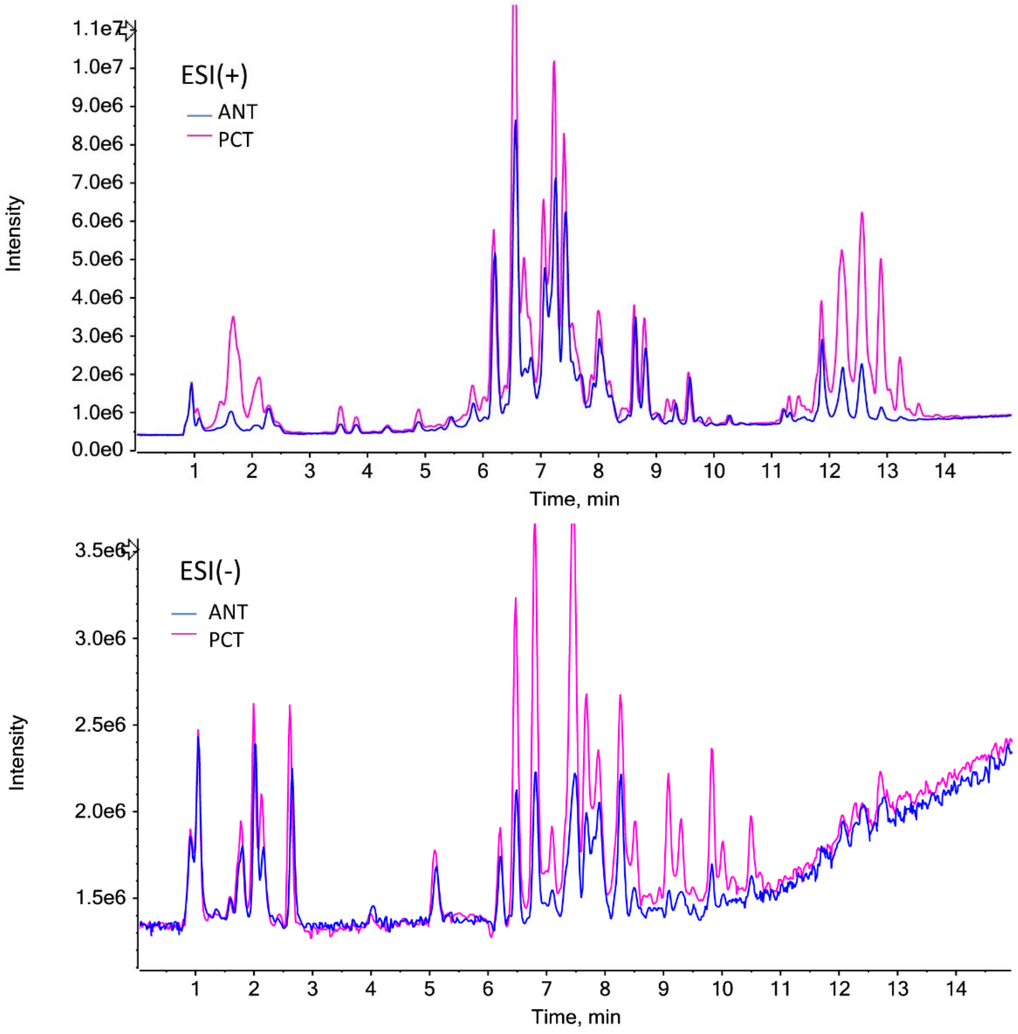

Fig. S2 Typical TICs of paired ANT and PCT samples by global lipidomics analysis in ESI positive and negative modes. upper panel: ESI positive; lower panel: ESI negative.

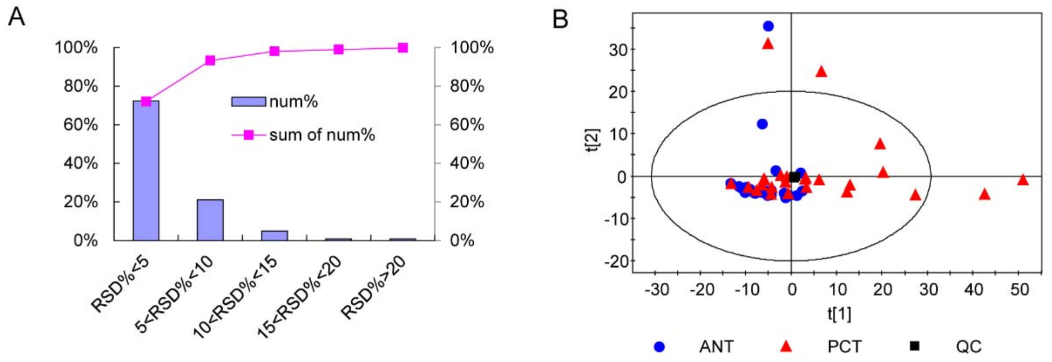

Fig. S3. Analytical precision and robustness evaluation of lipidomics analysis of discovery set. A, Coefficient of variation (CV) distribution of detected 350 lipid species in 7 QC samples. num%, percentage of lipid species within a defined CV range. sum of num%, accumulated percentage of lipid species below a defined CV criterion. CV is presented as relative standard deviation (RSD). B, Principal component analysis (PCA) score plot of lipidomic profile of 25 paired PCT-ANT tissue consisting of 347 lipid variables (RSD%<20) after unit variance (uv) scaling (R^2X=0.887, Q^2=0.576). Blue dot, red triangle and black box denote ANT, PCT and QC sample, respectively.

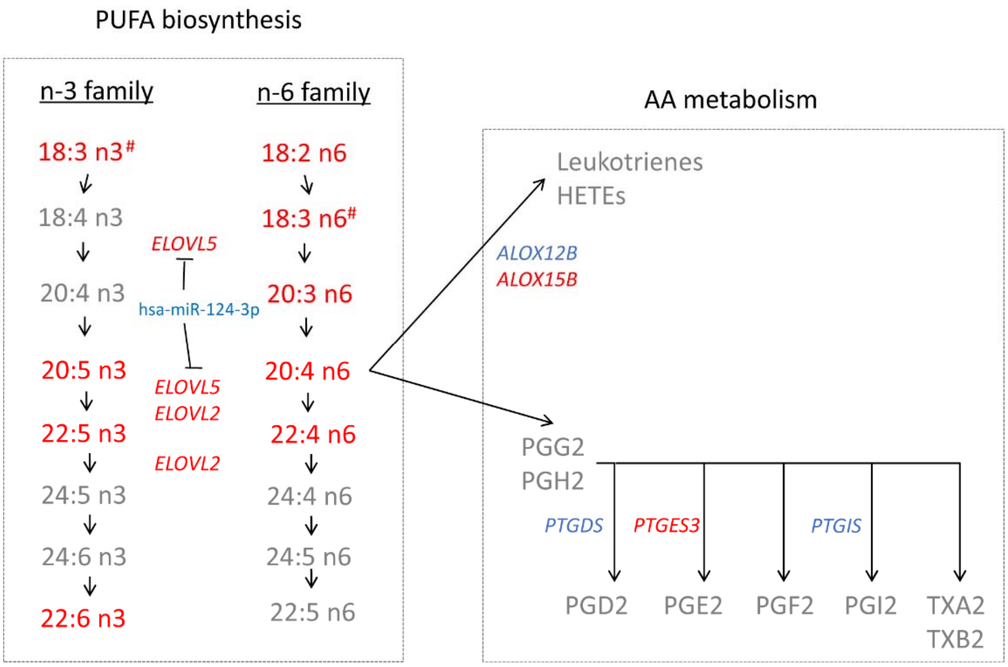

Fig. S4. Integrated pathway depicting PUFA biogenesis and metabolism. Red, blue, black and grey colored denote significantly increased, significantly decreased, unchanged, and undetected metabolite, or gene, or miRNA, respectively. In italics are gene names. #, isomers FFA 18:3 n3 and FFA 18:3 n6 are not distinguishable in applied lipidomics analysis, therefore increased level shown is their total amount. In PUFA biosynthesis, The rate-limiting gene *FADS2* (fatty acid desaturase, or Δ-6 desaturase) was not significantly changed. Hence we speculated that the biosynthesis of PUFA was unaffected. In arachidonic acid (AA) metabolism, genes *ALOX15B* (arachidonate 15-lipoxygenase, type B) and *PTGES3* (prostaglandin E synthase 3), involved in COX (cyclooxygenase) and LOX (lipoxygenase) pathways showed increased expression in PCa. HETE, hydroxyeicosatetraenoic acid; PGG2/H2/D2/E2/F2/I2, prostaglandin G2/H2/D2/E2/F2/I2; TXA2/B2, thromboxane A2/B2. Statistical significance was evaluated by Wilcoxon Signed-Rank test.

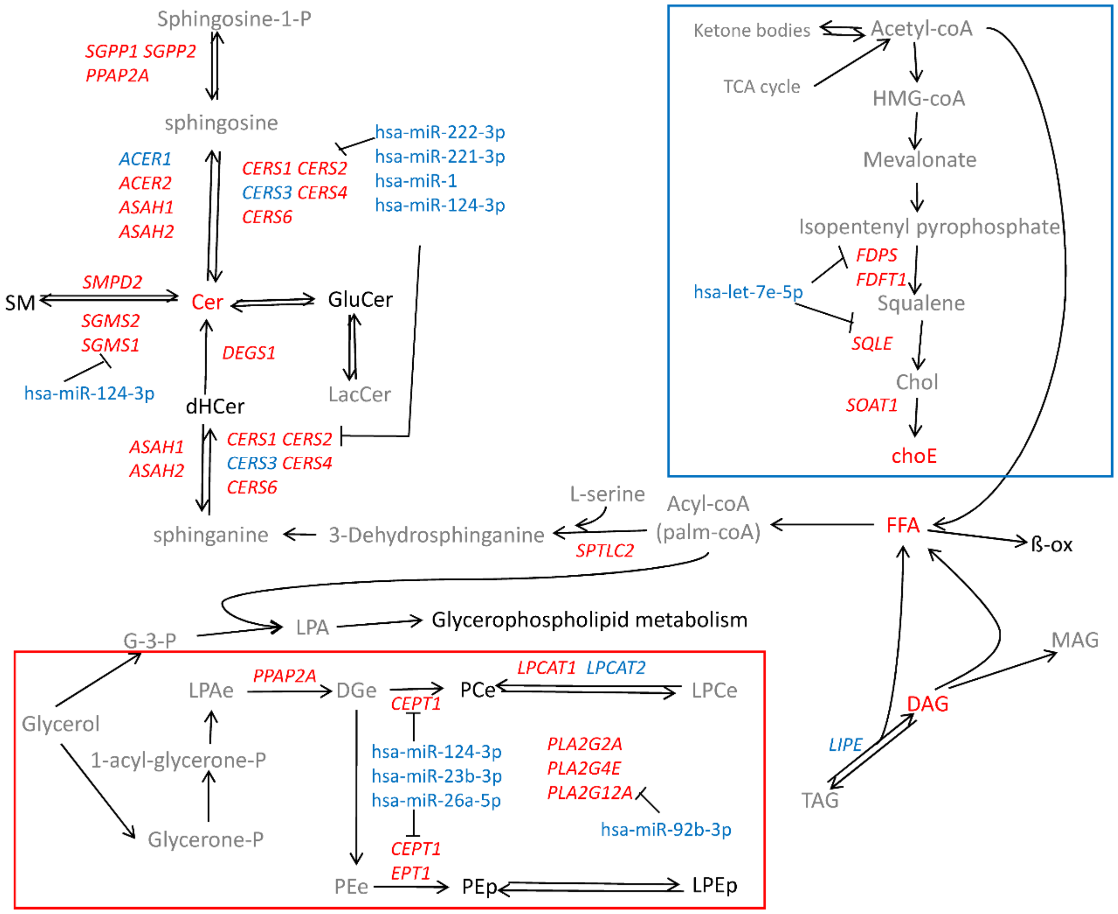

Fig. S5. Integrated pathway depicting lipid metabolism covering ether lipids, sphingolipids, glycerolipids and cholesterol. Red, blue, black and grey colored are significantly increased, significantly decreased, unchanged, and undetected metabolite, or gene, or miRNA, respectively. In italics are gene names. Red box and blue box indicate ether lipid and cholesterol metabolism, respectively. The characteristic remodeling pattern described in PL metabolism (Fig. 2B) was also observed in ether lipid pathway (red box) as revealed by expression alterations of a same set of genes in relation to PLA2 and LPLAT. It was consistent with the reduction of PUFA% and ether-linked chain% in PLs (Fig. 1E, 1F and supplementary Table S4) and reduction of a set of ether lipid-to-downstream metabolite-ratios, i.e., PE(18:0p/20:4)/LPE18:0p (PCT/ANT=0.62, p=0.005), PE(18:1p/20:4)/LPE18:1p (PCT/ANT =0.59, p=0.006), PE(18:0p/22:4)/LPE18:0p (PCT/ANT =0.65, p=0.019), PE(18:0p/22:6)/LPE18:0p (PCT/ANT =0.67, p=0.026). Depletion of ether-linked chain% may be due to the anti-oxidative nature of plasmalogens[1], in response to oxidative stress in PCa[2]. In Cholesterol biosynthesis (blue box), gene expression of *HMGCR* (HMG-CoA reductase), the major committed gene in cholesterol synthesis, was not changed. Therefore we speculated that cholesterol biosynthesis was unchanged. Statistical significance was evaluated by Wilcoxon Signed-Rank test.

Table S1 Detailed information of clinical characteristics of all participants enrolled in discovery and validation set.

No.	Preoperative PSA	Stage	Gleason Score	Metastasis*	sample tissue
			**discovery set**		
1	unknown	unknown	5+5	unknown	ANT, PCT
2	12.10	T2cN0M0	3+4	1	ANT, PCT
3	9.03	T3aN0M0	3+4	2	ANT, PCT
4	7.95	T2cN0M0	4+5	1	ANT, PCT
5	13.41	T2cN0M0	4+4	1	ANT, PCT
6	30.60	unknown	4+4	3	ANT, PCT
7	unknown	unknown	4+3	unknown	ANT, PCT
8	35.43	T2cN0M0	4+4	1	ANT, PCT
9	7.43	T2cN0M0	3+3	1	ANT, PCT
10	20.00	T2cN0M0	3+4	1	ANT, PCT
11	11.10	T2cN1M0	3+4	3	ANT, PCT
12	45.29	T3bN1M0	4+5	3	ANT, PCT
13	10.71	T3aN0M0	4+3	2	ANT, PCT
14	5.38	T2cN0M0	4+3	1	ANT, PCT
15	10.74	T2cN0M0	3+5	1	ANT, PCT
16	63.83	T3bN0M0	4+5	2	ANT, PCT
17	7.96	T2cN0M0	3+4	1	ANT, PCT
18	14.41	T2cN0M0	3+4	1	ANT, PCT
19	5.28	T2cN0M0	4+3	1	ANT, PCT
20	22.07	T2cN0M0	3+4	1	ANT, PCT
21	21.63	T2cN0M0	4+4	1	ANT, PCT
22	9.85	T3bN0M0	5+5	2	ANT, PCT
23	93.45	T2cN0M0	5+4	1	ANT, PCT
24	9.52	T2cN0M0	3+4	1	ANT, PCT
25	31.19	T2cN0M0	3+3	1	ANT, PCT
			**validation set**		
1	49.80	T2cN0M0	4+3	1	ANT, PCT
2	21.49	T3aN0M0	4+5	2	ANT, PCT
3	44.10	T2aN0M0	4+3	1	ANT, PCT
4	65.40	T2cN0M0	3+4	1	ANT, PCT
5	8.80	T2cN0M0	5+5	1	ANT, PCT
6	17.38	T2cN0M0	3+4	1	ANT, PCT
7	39.60	T3aN0M0	3+4	2	ANT, PCT
8	14.90	T2cN0M0	3+3	1	ANT, PCT
9	4.04	T2cN0M0	3+4	1	ANT, PCT
10	8.75	T2cN0M0	3+3	1	ANT, PCT
11	13.00	T2cN0M0	5+4	1	ANT, PCT
12	17.53	T4N0M0	3+4	2	ANT, PCT
13	13.91	T3bN1M0	5+4	3	ANT, PCT
14	14.91	T3bN0M0	5+4	2	ANT, PCT
15	22.75	T3bN0M0	4+3	2	ANT, PCT

16	22.00	T3bN0M0	5+4	2	ANT, PCT
17	44.38	T4N0M0	4+3	2	ANT, PCT
18	6.37	T4N0M0	3+4	2	ANT, PCT
19	100.00	T3bN0M0	4+3	2	ANT, PCT
20	12.14	T2cN0M0	3+4	1	ANT, PCT
21	12.80	T2cN0M0	3+4	1	ANT, PCT
22	52.58	T4N0M0	4+3	2	ANT, PCT
23	7.34	T2cN0M0	4+3	1	ANT, PCT
24	14.30	T3bN0M0	3+4	2	ANT, PCT
25	14.45	T2cN0M0	4+5	1	ANT, PCT
26	8.16	T2cN0M0	4+3	1	ANT, PCT
27	10.20	T3N1M0	5+5	1	ANT, PCT
28	11.47	T2bN0M0	3+4	1	ANT, PCT
29	25.09	T3aN0M0	4+5	2	ANT, PCT
30	25.68	T3aN0M0	3+4	2	ANT, PCT
31	8.68	T2bN0M0	3+3	1	ANT, PCT
32	28.00	T3bN0M0	4+5	2	ANT, PCT
33	25.00	T2cN0M0	4+5	1	ANT, PCT
34	16.00	T2bN0M0	3+4	1	ANT, PCT
35	12.30	T2bN0M0	3+4	1	ANT, PCT
36	11.22	T2cN0M0	3+4	1	ANT, PCT
37	4.48	T2bN0M0	3+3	1	ANT, PCT
38	62.00	T2cN0M0	4+3	1	ANT, PCT
39	43.50	T2cN0M0	4+3	1	ANT, PCT
40	18.26	T2bN0M0	3+4	1	ANT, PCT
41	9.08	T2bN0M0	3+4	1	ANT, PCT
42	17.30	T2cN0M0	4+4	1	ANT, PCT
43	17.87	T2bN0M0	3+4	1	ANT, PCT
44	41.39	T3aN1M0	5+4	3	ANT, PCT
45	30.31	T3bN0M0	3+4	2	ANT, PCT
46	15.50	T2bN0M0	3+4	1	ANT, PCT
47	26.44	T2bN0M0	3+3	1	ANT, PCT
48	0.63	T2cN0M0	4+3	1	ANT, PCT
49	40.38	T3aN0M0	4+3	2	ANT, PCT
50	13.10	T2cN0M0	5+3	1	ANT, PCT
51	16.66	T2bN0M0	3+3	1	ANT, PCT
B1	23.68				BPH
B2	16.76				BPH
B3	1.46				BPH
B4	11.04				BPH
B5	42.73				BPH
B6	5.66				BPH
B7	1.79				BPH
B8	3.09				BPH
B9	0.53				BPH
B10	10.60				BPH

B11	unknown	BPH
B12	unknown	BPH
B13	unknown	BPH
B14	0.53	BPH
B15	0.99	BPH
B16	1.09	BPH
B17	1.17	BPH
B18	1.62	BPH
B19	0.96	BPH

*, 1=localized, 2=locally advanced, 3=metastatic

Table S2 Comprehensive list of identified 350 lipids in prostate tissue. Unique chain compositions were assigned for approximately 99% of sphingolipids and 97% of phospholipids (calculated as abundance %) based on high resolution MS/MS. PCT/ANT, folds change of PCT vs. ANT, calculated as ratio of mean value of PCT vs. ANT. Statistical significance was determined by Wilcoxon Signed-Rank test.

lipid	composition*	adduct in ESI(+)	adduct in ESI(-)	polarity for quantitation	PCT vs ANT (discovery set) PCT/ANT	*p* value
CE 18:1		M+NH4		positive	11.78	0.000
CE 20:1		M+NH4		positive	45.48	0.000
CE 22:6		M+NH4		positive	10.48	0.000
CE 24:4		M+NH4		positive	24.86	0.000
CE 24:5		M+NH4		positive	35.46	0.000
CE 28:5		M+NH4		positive	27.96	0.000
Cer 34:1;2	Cer(d18:1/16:0)	M+H		positive	1.46	0.122
Cer 34:2;2	unknown	M+H		positive	4.44	0.000
Cer 36:1;2	Cer(d18:1/18:0)	M+H		positive	2.24	0.007
Cer 38:1;2	Cer(d18:1/20:0)	M+H		positive	2.41	0.001
Cer 38:2;2	Cer(d18:2/20:0)	M+H		positive	11.38	0.000
Cer 40:1;2	Cer(d18:1/22:0)	M+H		positive	2.52	0.002
Cer 40:2;2	Cer(d18:1/22:1)	M+H		positive	5.99	0.000
Cer 41:1;2	Cer(d18:1/23:0)	M+H		positive	3.32	0.001
Cer 42:1;2	Cer(d18:1/24:0)	M+H		positive	1.50	0.221
Cer 42:2;2	Cer(d18:1/24:1)	M+H		positive	1.47	0.158
DAG 34:2		M+NH4		positive	1.83	0.011
DAG 36:1		M+NH4		positive	2.97	0.000
DAG 36:2		M+NH4		positive	2.27	0.001
DAG 36:3		M+NH4		positive	2.04	0.002
DAG 36:4		M+NH4		positive	1.39	0.143
DAG 38:2		M+NH4		positive	6.54	0.000
DAG 38:4		M+NH4		positive	0.79	0.032
FFA14:0			M-H	negative	1.33	0.017
FFA15:0			M-H	negative	1.06	0.326
FFA16:0			M-H	negative	1.20	0.109
FFA16:1			M-H	negative	1.70	0.004
FFA17:0			M-H	negative	1.26	0.023
FFA18:0			M-H	negative	1.14	0.264
FFA18:1			M-H	negative	2.51	0.000
FFA18:2			M-H	negative	1.83	0.008
FFA18:3			M-H	negative	1.41	0.035
FFA19:0			M-H	negative	1.73	0.000
FFA20:0			M-H	negative	1.76	0.001
FFA20:1			M-H	negative	5.46	0.000
FFA20:2			M-H	negative	3.90	0.000
FFA20:3			M-H	negative	4.00	0.000
FFA20:4			M-H	negative	2.61	0.000

FFA20:5			M-H	negative	2.49	0.002
FFA22:0			M-H	negative	2.10	0.000
FFA22:1			M-H	negative	3.25	0.000
FFA22:2			M-H	negative	4.43	0.000
FFA22:3			M-H	negative	5.39	0.000
FFA22:4			M-H	negative	2.09	0.020
FFA22:5			M-H	negative	3.06	0.001
FFA22:6			M-H	negative	3.51	0.000
HexCer 34:1;2	HexCer(d18:1/16:0)	M+H		positive	1.12	0.397
HexCer 34:1;3	HexCer(d18:1/16:0)-OH	M+H		positive	4.57	0.001
HexCer 36:1;2	HexCer(d18:1/18:0)	M+H		positive	1.71	0.443
HexCer 38:1;2	HexCer(d18:1/20:0)	M+H		positive	2.52	0.211
HexCer 40:1;2	HexCer(d18:1/22:0)	M+H		positive	2.89	0.042
HexCer 40:2;2	HexCer(d18:1/22:1)	M+H		positive	10.22	0.000
HexCer 41:1;2	HexCer(d18:1/23:0)	M+H		positive	4.45	0.000
HexCer 42:1;2	HexCer(d18:1/24:0)	M+H		positive	1.88	0.677
LPC 14:0		M+H	M+AcO	positive	4.54	0.002
LPC 16:0		M+H	M+AcO	positive	2.48	0.083
LPC 17:0		M+H	M+AcO	positive	2.33	0.004
LPC 18:0		M+H	M+AcO	positive	1.82	0.231
LPC 18:1		M+H	M+AcO	positive	3.05	0.042
LPC 18:2		M+H	M+AcO	positive	1.54	0.427
LPC 20:0		M+H	M+AcO	positive	3.15	0.004
LPC 20:1		M+H	M+AcO	positive	3.36	0.074
LPC 20:2		M+H	M+AcO	positive	2.59	0.115
LPC 20:4		M+H	M+AcO	positive	0.85	0.476
LPE 16:0		M+H	M-H	positive	2.04	0.696
LPE 17:0		M+H	M-H	positive	1.51	0.042
LPE 18:0		M+H	M-H	positive	2.40	0.115
LPE 18:1		M+H	M-H	positive	4.04	0.061
LPE 18:2		M+H	M-H	positive	3.59	0.032
LPE 20:0		M+H	M-H	positive	4.68	0.000
LPE 20:1		M+H	M-H	positive	3.95	0.065
LPE 20:2		M+H	M-H	positive	3.67	0.061
LPE 20:4		M+H	M-H	positive	1.12	0.696
LPE 22:6		M+H	M-H	positive	1.69	0.058
LPE P-16:0		M+H	M-H	positive	1.91	0.264
LPE P-18:0		M+H	M-H	positive	1.76	0.382
LPE P-18:1		M+H	M-H	positive	2.74	0.143
LPI 18:0		$M+NH_4$	M-H	negative	2.36	0.001
LPS 18:0		M+H	M-H	positive	3.52	0.001
PA 36:1	PA(18:0/18:1)	$M+NH_4$	M-H	negative	1.23	0.493
PC 28:0	unknown	M+H	M+AcO	positive	5.39	0.000
PC 30:1	unknown	M+H	M+AcO	positive	3.68	0.000
PC 31:0	PC(15:0/16:0)	M+H	M+AcO	positive	1.25	0.242
PC 32:0	PC(16:0/16:0)	M+H	M+AcO	positive	0.94	0.459

PC 32:1	PC(14:0/18:1)	M+H	M+AcO	positive	2.34	0.001
PC 32:2	PC(16:1/16:1)	M+H	M+AcO	positive	2.30	0.003
PC 33:0	PC(16:0/17:0)	M+H	M+AcO	positive	2.54	0.000
PC 33:1	PC(16:0/17:1)	M+H	M+AcO	positive	2.57	0.000
PC 33:2	unknown	M+H	M+AcO	positive	3.14	0.000
PC 34:0	PC(16:0/18:0)	M+H	M+AcO	positive	1.22	0.300
PC 34:1	PC(16:0/18:1)	M+H	M+AcO	positive	3.19	0.000
PC 34:2	PC(16:0/18:2)	M+H	M+AcO	positive	1.57	0.065
PC 34:3	PC(16:0/18:3)	M+H	M+AcO	positive	1.69	0.019
PC 34:4	PC(20:4/14:0)	M+H	M+AcO	positive	2.06	0.002
PC 35:1	PC(17:0/18:1)	M+H	M+AcO	positive	3.72	0.000
PC 35:2	PC(18:2/17:0)	M+H	M+AcO	positive	1.75	0.004
PC 35:3	unknown	M+H	M+AcO	positive	1.73	0.007
PC 35:4	PC(20:4/15:0)	M+H	M+AcO	positive	1.12	0.493
PC 36:0	PC(18:0/18:0)	M+H	M+AcO	positive	1.75	0.007
PC 36:1	PC(18:0/18:1)	M+H	M+AcO	positive	2.25	0.001
PC 36:2	PC(18:1/18:1)	M+H	M+AcO	positive	1.68	0.058
PC 36:3	PC(18:1/18:2)	M+H	M+AcO	positive	1.97	0.004
PC 36:4	PC(16:0/20:4)	M+H	M+AcO	positive	1.16	0.861
PC 36:5	PC(16:0/20:5)	M+H	M+AcO	positive	1.10	0.600
PC 37:1	unknown	M+H	M+AcO	positive	4.12	0.000
PC 37:2	unknown	M+H	M+AcO	positive	3.18	0.000
PC 37:4	PC(20:4/17:0)	M+H	M+AcO	positive	1.23	0.326
PC 38:1	unknown	M+H	M+AcO	positive	2.84	0.001
PC 38:2	PC(18:0/20:2)	M+H	M+AcO	positive	3.06	0.001
PC 38:3	PC(18:0/20:3)	M+H	M+AcO	positive	1.43	0.093
PC 38:4	PC(18:0/20:4)	M+H	M+AcO	positive	0.97	0.476
PC 38:5	PC(16:0/22:5)	M+H	M+AcO	positive	1.50	0.088
PC 38:6	PC(16:0/22:6)	M+H	M+AcO	positive	1.40	0.166
PC 40:3	PC(20:2/20:1)	M+H	M+AcO	positive	2.28	0.005
PC 40:4	PC(18:0/22:4)/PC(20:3/20:1)	M+H	M+AcO	positive	1.39	0.326
PC 40:5	PC(22:5/18:0)	M+H	M+AcO	positive	1.46	0.054
PC 40:6	PC(18:0/22:6)	M+H	M+AcO	positive	1.21	0.619
PC O-30:0	unknown	M+H	M+AcO	positive	1.42	0.276
PC O-30:1	unknown	M+H	M+AcO	positive	2.04	0.007
PC O-32:0	PC(16:0e/16:0)	M+H	M+AcO	positive	0.91	0.545
PC O-32:1	PC(16:1e/16:0)	M+H	M+AcO	positive	1.15	0.757
PC O-34:0	PC(18:0e/16:0)	M+H	M+AcO	positive	0.99	0.677
PC O-34:1	PC(18:1e/16:0)	M+H	M+AcO	positive	1.06	0.925
PC O-34:2	unknown	M+H	M+AcO	positive	0.94	0.925
PC O-34:3	PC(18:3e/16:0)/PC(16:1e/18:2)	M+H	M+AcO	positive	1.30	0.353
PC O-36:3	unknown	M+H	M+AcO	positive	1.10	0.968
PC O-36:4	PC(16:0e/20:4)	M+H	M+AcO	positive	0.94	0.326
PC O-36:5	PC(16:1e/20:4)	M+H	M+AcO	positive	1.08	0.968
PC O-38:3	unknown	M+H	M+AcO	positive	2.15	0.002
PC O-38:5	PC(18:1e/20:4)	M+H	M+AcO	positive	0.91	0.313

PC O-38:7	unknown	M+H	M+AcO	positive	1.34	0.581
PC O-42:5	unknown	M+H	M+AcO	positive	1.27	0.527
PC O-44:6	unknown	M+H	M+AcO	positive	0.71	0.201
PE 32:0	PE(16:0/16:0)	M+H	M-H	positive	0.97	0.276
PE 32:1	PE(16:0/16:1)	M+H	M-H	positive	3.86	0.000
PE 33:1	PE(15:0/18:1)	M+H	M-H	positive	2.97	0.000
PE 34:1	PE(16:0/18:1)	M+H	M-H	positive	3.11	0.000
PE 34:2	PE(16:0/18:2)	M+H	M-H	positive	2.45	0.001
PE 34:3	unknown	M+H	M-H	positive	3.19	0.001
PE 35:1	PE(16:0/19:1)	M+H	M-H	positive	3.20	0.000
PE 35:2	unknown	M+H	M-H	positive	2.42	0.000
PE 36:1	PE(18:1/18:0)	M+H	M-H	positive	2.21	0.000
PE 36:2	PE(18:2/18:0)	M+H	M-H	positive	3.10	0.000
PE 36:3	PE(18:2/18:1)	M+H	M-H	positive	2.83	0.001
PE 36:4	PE(16:0/20:4)	M+H	M-H	positive	1.45	0.211
PE 36:5	PE(20:4/16:1)	M+H	M-H	positive	1.80	0.009
PE 37:2	PE(18:1/19:1)	M+H	M-H	positive	2.83	0.000
PE 38:1	PE(18:1/20:0)	M+H	M-H	positive	2.65	0.001
PE 38:2	PE(18:1/20:1)	M+H	M-H	positive	3.12	0.000
PE 38:3	PE(18:0/20:3)	M+H	M-H	positive	1.71	0.009
PE 38:4	PE(18:0/20:4)	M+H	M-H	positive	1.24	0.242
PE 38:5	PE(18:1/20:4)	M+H	M-H	positive	1.75	0.015
PE 38:6	PE(16:0/22:6)	M+H	M-H	positive	1.71	0.017
PE 40:1	PE(18:1/22:0)	M+H	M-H	positive	2.78	0.000
PE 40:2	PE(18:1/22:1)	M+H	M-H	positive	2.91	0.000
PE 40:3	PE(18:1/22:2)	M+H	M-H	positive	2.21	0.019
PE 40:4	PE(18:0/22:4)	M+H	M-H	positive	1.09	0.968
PE 40:5	PE(18:0/22:5)	M+H	M-H	positive	3.33	0.002
PE 40:6	PE(18:0/22:6)	M+H	M-H	positive	2.17	0.002
PE 40:7	PE(22:6/18:1)	M+H	M-H	positive	2.24	0.001
PE 42:1	PE(18:1/24:0)	M+H	M-H	positive	1.75	0.015
PE 42:4	PE(20:4/22:0)	M+H	M-H	positive	3.05	0.004
PE 42:5	PE(20:4/22:1)	M+H	M-H	positive	2.22	0.009
PE P-32:0	PE(16:0p/16:0)	M+H	M-H	positive	0.97	0.427
PE P-32:1	PE(16:0p/16:1)	M+H	M-H	positive	3.98	0.001
PE P-34:0	PE(18:0p/16:0)	M+H	M-H	positive	0.93	0.545
PE P-34:1	PE(16:0p/18:1)	M+H	M-H	positive	2.53	0.008
PE P-34:2	PE(16:0p/18:2)	M+H	M-H	positive	1.93	0.069
PE P-35:1	PE(17:0p/18:1)	M+H	M-H	positive	2.21	0.001
PE P-36:1	PE(18:0p/18:1)	M+H	M-H	positive	1.67	0.122
PE P-36:2	PE(18:1p/18:1)/PE(16:0p/20:2)	M+H	M-H	positive	1.89	0.109
PE P-36:3	PE(16:0p/20:3)	M+H	M-H	positive	1.95	0.025
PE P-36:4	PE(16:0p/20:4)	M+H	M-H	positive	1.26	0.382
PE P-36:5	PE(16:0p/20:5)	M+H	M-H	positive	1.39	0.353
PE P-37:4	PE(17:0p/20:4)	M+H	M-H	positive	1.02	0.819
PE P-38:2	PE(18:1p/20:1)	M+H	M-H	positive	2.11	0.051

PE P-38:3	PE(18:0p/20:3)	M+H	M-H	positive	1.49	0.231
PE P-38:4	PE(18:0p/20:4)	M+H	M-H	positive	0.85	0.150
PE P-38:5	PE(18:1p/20:4)	M+H	M-H	positive	1.07	0.677
PE P-38:6	PE(16:0p/22:6)	M+H	M-H	positive	1.42	0.313
PE P-40:4	PE(18:0p/22:4)	M+H	M-H	positive	0.84	0.088
PE P-40:5	PE(18:1p/22:4)/PE(18:2p/22:3)	M+H	M-H	positive	1.07	0.989
PE P-40:6	PE(18:0p/22:6)	M+H	M-H	positive	1.01	0.716
PG 34:1	PG(16:0/18:1)	$M+NH_4$	M-H	negative	1.72	0.010
PG 36:1	PG(18:0/18:1)	$M+NH_4$	M-H	negative	0.99	0.737
PG 36:2	PG(18:1/18:1)	$M+NH_4$	M-H	negative	4.35	0.000
PG 36:3	PG(18:2/18:1)	$M+NH_4$	M-H	negative	2.87	0.002
PG 36:4	PG(18:2/18:2)	$M+NH_4$	M-H	negative	2.11	0.065
PG 38:3	PG(18:1/20:2)/PG(18:2/20:1)	$M+NH_4$	M-H	negative	6.07	0.000
PG 40:6	PG(22:5/18:1)/PG(22:6/18:0)	$M+NH_4$	M-H	negative	1.94	0.109
PG 40:7	PG(18:1/22:6)	$M+NH_4$	M-H	negative	1.34	0.882
PG 40:8	PG(22:6/18:2)	$M+NH_4$	M-H	negative	1.17	0.040
PG 44:12	PG(22:6/22:6)	$M+NH_4$	M-H	negative	0.65	0.006
PI 32:0	PI(16:0/16:0)	$M+NH_4$	M-H	negative	2.79	0.003
PI 34:0	PI(16:0/18:0)	$M+NH_4$	M-H	negative	3.68	0.000
PI 34:1	PI(16:0/18:1)	$M+NH_4$	M-H	negative	2.24	0.004
PI 34:2	PI(16:0/18:2)	$M+NH_4$	M-H	negative	1.54	0.201
PI 36:1	PI(18:1/18:0)	$M+NH_4$	M-H	negative	4.97	0.000
PI 36:2	PI(18:1/18:1)	$M+NH_4$	M-H	negative	2.61	0.001
PI 36:3	PI(16:0/20:3)	$M+NH_4$	M-H	negative	1.76	0.019
PI 36:4	PI(16:0/20:4)	$M+NH_4$	M-H	negative	1.02	0.510
PI 37:4	PI(17:0/20:4)	$M+NH_4$	M-H	negative	1.18	0.264
PI 38:2	PI(18:0/20:2)	$M+NH_4$	M-H	negative	6.47	0.000
PI 38:3	PI(18:0/20:3)	$M+NH_4$	M-H	negative	3.29	0.000
PI 38:4	PI(18:0/20:4)	$M+NH_4$	M-H	negative	1.07	0.904
PI 38:5	PI(18:1/20:4)	$M+NH_4$	M-H	negative	1.25	0.657
PI 38:6	PI(16:0/22:6)	$M+NH_4$	M-H	negative	1.69	0.104
PI 40:6	PI(18:0/22:6)	$M+NH_4$	M-H	negative	3.53	0.000
PS 34:1	PS(16:0/18:1)	M+H	M-H	positive	1.36	0.300
PS 34:2	PS(16:0/18:2)	M+H	M-H	positive	1.38	0.382
PS 36:1	PS(18:0/18:1)	M+H	M-H	positive	1.29	0.545
PS 36:2	PS(18:0/18:2)	M+H	M-H	positive	1.89	0.045
PS 36:3	PS(16:0/20:3)	M+H	M-H	positive	1.66	0.093
PS 36:4	unknown	M+H	M-H	positive	0.86	0.253
PS 38:1	PS(18:1/20:0)	M+H	M-H	positive	2.01	0.010
PS 38:2	PS(18:1/20:1)	M+H	M-H	positive	1.90	0.023
PS 38:3	PS(18:0/20:3)	M+H	M-H	positive	1.42	0.174
PS 38:4	PS(18:0/20:4)	M+H	M-H	positive	0.94	0.476
PS 38:6	PS(16:0/22:6)	M+H	M-H	positive	0.65	0.009
PS 40:1	PS(18:1/22:0)	M+H	M-H	positive	2.09	0.003
PS 40:2	PS(18:1/22:1)	M+H	M-H	positive	1.96	0.004
PS 40:4	PS(18:0/22:4)	M+H	M-H	positive	0.96	0.510

PS 40:5	PS(18:0/22:5)	M+H	M-H	positive	1.61	0.078
PS 40:6	PS(18:0/22:6)	M+H	M-H	positive	1.38	0.192
PS 42:1	unknown	M+H	M-H	positive	1.70	0.020
PS 42:2	unknown	M+H	M-H	positive	1.63	0.042
PS 42:6	unknown	M+H	M-H	positive	2.41	0.002
PS 44:6	unknown	M+H	M-H	positive	2.06	0.001
PS O-46:0	unknown	M+H	M-H	positive	1.50	0.882
SM 31:1;2	SM(d18:1/13:0)	M+H	M+AcO	positive	1.95	0.019
SM 32:1;2	SM(d18:1/14:0)	M+H	M+AcO	positive	1.35	0.253
SM 32:1;3	SM(d18:1/14:0-OH)	M+H	M+AcO	positive	2.82	0.021
SM 33:1;2	SM(d18:1/15:0)	M+H	M+AcO	positive	1.09	0.778
SM 34:0;2	SM(d18:0/16:0)	M+H	M+AcO	positive	1.20	0.510
SM 34:1;2	SM(d18:1/16:0)	M+H	M+AcO	positive	1.11	0.778
SM 34:1;3	SM(d18:1/16:0)-OH	M+H	M+AcO	positive	1.59	0.037
SM 34:2;2	SM(d18:1/16:1)	M+H	M+AcO	positive	1.23	0.288
SM 35:1;2	SM(d18:1/17:0)	M+H	M+AcO	positive	1.31	0.150
SM 36:1;2	SM(d18:1/18:0)	M+H	M+AcO	positive	1.68	0.051
SM 36:2;2	unknown	M+H	M+AcO	positive	1.37	0.078
SM 38:0;2	SM(d18:0/20:0)	M+H	M+AcO	positive	4.18	0.143
SM 38:1;2	SM(d18:1/20:0)	M+H	M+AcO	positive	2.52	0.005
SM 38:2;2	SM(d18:1/20:1)	M+H	M+AcO	positive	4.13	0.000
SM 39:1;2	SM(d18:1/21:0)	M+H	M+AcO	positive	3.96	0.000
SM 40:0;3	SM(d18:0/22:0)-OH	M+H	M+AcO	positive	2.89	0.001
SM 40:1;2	SM(d18:1/22:0)	M+H	M+AcO	positive	1.98	0.005
SM 40:2;2	SM(d18:1/22:1)	M+H	M+AcO	positive	2.58	0.001
SM 40:3;2	SM(d18:1/22:2)	M+H	M+AcO	positive	1.39	0.109
SM 41:1;2	SM(d18:1/23:0)	M+H	M+AcO	positive	2.44	0.000
SM 41:2;2	SM(d18:1/23:1)/SM(18:2/23:0)	M+H	M+AcO	positive	1.61	0.015
SM 42:0;3	SM(d18:0/24:0)-OH	M+H	M+AcO	positive	1.35	0.035
SM 42:1;2	SM(d18:1/24:0)	M+H	M+AcO	positive	1.29	0.231
SM 42:1;3	SM(d18:1/24:0)-OH	M+H	M+AcO	positive	2.54	0.001
SM 42:2;2	SM(d18:1/24:1)/SM(d18:2/24:0)	M+H	M+AcO	positive	1.00	0.840
SM 42:3;2	SM(d18:1/24:2)	M+H	M+AcO	positive	0.89	0.339
SM 43:2;2	SM(d18:1/25:1)	M+H	M+AcO	positive	1.03	0.677
SM 44:1;2	SM(d18:1/26:0)	M+H	M+AcO	positive	0.70	0.023
SM 44:2;2	SM(d18:1/26:1)	M+H	M+AcO	positive	0.77	0.045
SM 44:3;2	SM(d18:1/26:2)	M+H	M+AcO	positive	0.88	0.192
TAG 40:0		$M+NH_4$		positive	2.10	0.211
TAG 40:1		$M+NH_4$		positive	3.50	0.183
TAG 42:0		$M+NH_4$		positive	2.14	0.288
TAG 42:1		$M+NH_4$		positive	2.43	0.174
TAG 44:0		$M+NH_4$		positive	2.31	0.042
TAG 44:1		$M+NH_4$		positive	1.90	0.313
TAG 44:2		$M+NH_4$		positive	1.57	0.778
TAG 45:1		$M+NH_4$		positive	1.30	0.069
TAG 46:0		$M+NH_4$		positive	2.77	0.002

TAG 46:1	$M+NH_4$	positive	1.70	0.158
TAG 46:2	$M+NH_4$	positive	1.29	0.527
TAG 46:3	$M+NH_4$	positive	1.25	0.840
TAG 47:0	$M+NH_4$	positive	1.50	0.009
TAG 47:1	$M+NH_4$	positive	1.46	0.040
TAG 48:0	$M+NH_4$	positive	2.66	0.002
TAG 48:1	$M+NH_4$	positive	1.67	0.104
TAG 48:2	$M+NH_4$	positive	1.12	0.563
TAG 48:3	$M+NH_4$	positive	1.09	0.882
TAG 48:4	$M+NH_4$	positive	0.98	0.716
TAG 49:0	$M+NH_4$	positive	2.14	0.001
TAG 49:1	$M+NH_4$	positive	1.36	0.054
TAG 49:2	$M+NH_4$	positive	1.09	0.300
TAG 49:3	$M+NH_4$	positive	1.08	0.677
TAG 50:0	$M+NH_4$	positive	3.26	0.001
TAG 50:1	$M+NH_4$	positive	1.35	0.104
TAG 50:2	$M+NH_4$	positive	0.87	0.527
TAG 50:3	$M+NH_4$	positive	0.78	0.946
TAG 50:4	$M+NH_4$	positive	0.81	0.925
TAG 50:5	$M+NH_4$	positive	1.12	0.798
TAG 51:0	$M+NH_4$	positive	3.62	0.000
TAG 51:1	$M+NH_4$	positive	1.63	0.023
TAG 51:2	$M+NH_4$	positive	0.95	0.253
TAG 51:3	$M+NH_4$	positive	0.68	0.657
TAG 51:4	$M+NH_4$	positive	0.57	0.696
TAG 52:0	$M+NH_4$	positive	4.47	0.000
TAG 52:1	$M+NH_4$	positive	1.73	0.028
TAG 52:2	$M+NH_4$	positive	0.76	0.563
TAG 52:3	$M+NH_4$	positive	0.47	0.989
TAG 52:4	$M+NH_4$	positive	0.35	0.412
TAG 52:5	$M+NH_4$	positive	0.43	0.397
TAG 52:6	$M+NH_4$	positive	1.03	0.882
TAG 53:2	$M+NH_4$	positive	1.03	0.201
TAG 53:3	$M+NH_4$	positive	0.61	0.581
TAG 53:4	$M+NH_4$	positive	0.50	0.819
TAG 53:5	$M+NH_4$	positive	0.64	0.778
TAG 54:0	$M+NH_4$	positive	4.63	0.000
TAG 54:1	$M+NH_4$	positive	3.55	0.001
TAG 54:2	$M+NH_4$	positive	1.23	0.061
TAG 54:3	$M+NH_4$	positive	0.63	0.427
TAG 54:4	$M+NH_4$	positive	0.41	0.757
TAG 54:5	$M+NH_4$	positive	0.37	0.840
TAG 54:6	$M+NH_4$	positive	0.44	0.925
TAG 54:7	$M+NH_4$	positive	0.65	0.677
TAG 54:8	$M+NH_4$	positive	1.25	0.989
TAG 55:1	$M+NH_4$	positive	7.81	0.000

TAG 55:2		$M+NH_4$		positive	3.22	0.003
TAG 55:3		$M+NH_4$		positive	1.29	0.048
TAG 55:7		$M+NH_4$		positive	0.79	0.476
TAG 56:0		$M+NH_4$		positive	3.89	0.000
TAG 56:1		$M+NH_4$		positive	6.40	0.000
TAG 56:2		$M+NH_4$		positive	3.54	0.002
TAG 56:3		$M+NH_4$		positive	2.05	0.021
TAG 56:4		$M+NH_4$		positive	1.30	0.088
TAG 56:5		$M+NH_4$		positive	1.06	0.192
TAG 56:6		$M+NH_4$		positive	1.01	0.367
TAG 56:7		$M+NH_4$		positive	1.15	0.476
TAG 56:8		$M+NH_4$		positive	1.20	0.861
TAG 56:9		$M+NH_4$		positive	1.37	0.563
TAG 58:0		$M+NH_4$		positive	3.52	0.000
TAG 58:1		$M+NH_4$		positive	6.25	0.000
TAG 58:10		$M+NH_4$		positive	1.28	0.600
TAG 58:2		$M+NH_4$		positive	4.03	0.001
TAG 58:3		$M+NH_4$		positive	2.35	0.006
TAG 58:4		$M+NH_4$		positive	2.61	0.005
TAG 58:5		$M+NH_4$		positive	2.71	0.009
TAG 58:6		$M+NH_4$		positive	2.10	0.035
TAG 58:7		$M+NH_4$		positive	1.45	0.143
TAG 58:8		$M+NH_4$		positive	1.49	0.276
TAG 58:9		$M+NH_4$		positive	1.25	0.443
TAG 60:1		$M+NH_4$		positive	8.32	0.000
TAG 60:2		$M+NH_4$		positive	5.06	0.002
TAG 60:3		$M+NH_4$		positive	2.52	0.014
TAG 60:4		$M+NH_4$		positive	2.68	0.009
TAG 60:5		$M+NH_4$		positive	4.48	0.002
TAG 60:6		$M+NH_4$		positive	5.16	0.004
TAG 60:8		$M+NH_4$		positive	3.01	0.040
TAG 60:9		$M+NH_4$		positive	2.87	0.074
TG O-50:1		$M+NH_4$		positive	3.54	0.002
HexCer 34:2;2[#]	HexCer(d18:1/16:1)	M+H		positive	/	/
FFA 21:0[#]			M-H	negative	/	/
PE 37:1[#]	unknown	M+H	M-H	positive	/	/

*, chain composition presented for phospholipids does not indicate sterospecificity;
#, these three lipid species presented higher than 20% of RSD values of QC samples and were excluded for further analysis.

Table S3 Comparison of lipid abundance and its composition in paired ANT and PCT tissue. Lipid abundance was quantified by normalization to respective internal standards. Lipid composition was calculated as percentage of (sub)classes in entire lipidome. PCT/ANT, folds change of PCT vs. ANT, calculated as ratio of mean value of PCT vs. ANT. Statistical significance was determined by Wilcoxon Signed-Rank test. n.s., not significant.

lipid	abundance		composition (%)	
(sub)class	PCT/ANT	*p* value	PCT/ANT	*p* value
FFA	1.65	<0.01	1.00	n.s.
Cer	2.03	<0.01	1.11	n.s.
HexCer	2.31	n.s.	1.14	n.s.
SM	1.38	n.s.	0.82	<0.01
LPC	2.35	n.s.	1.19	n.s.
LPE	2.60	n.s.	1.21	n.s.
LPE-P	2.07	n.s.	1.06	n.s.
diacyl-PC	1.93	<0.01	1.11	<0.01
PC-O	1.04	n.s.	0.65	<0.001
diacyl-PE	2.18	<0.001	1.24	<0.05
PE-P	1.26	n.s.	0.74	<0.01
PG	1.73	<0.05	1.04	n.s.
PI	1.81	<0.05	1.03	n.s.
PS	1.42	n.s.	0.84	<0.01
DAG	1.72	<0.01	1.11	n.s.
TAG	0.75	n.s.	0.73	n.s.
CE	15.87	<0.001	9.26	<0.001

Table S4 Composition of fatty acids or radyl residues in various lipid pools including FFA and 5 PL categories (PC, PE, PI, PS, PG). Percentage of SFA-, MUFA-, PUFA-acyl chains and ether-linked chains were calculated based on identified acyl/alkyl/alkenyl chain composition of individual PL species. Mean, mean value; SD, standard deviation; FC, folds change of PCT vs. ANT, calculated as ratio of mean value of PCT vs. ANT; Statistical significance was determined by Wilcoxon Signed-Rank test.

lipid pool	SFA						MUFA						PUFA						ether						missing*
	ANT		PCT		FC	*p*	ANT		PCT		FC	*p*	ANT		PCT		FC	*p*	ANT		PCT		FC	*p*	
	mean	SD	mean	SD			mean	SD	mean	SD			mean	SD	mean	SD			mean	SD	mean	SD			
FFA	65.5%	6.1%	51.0%	11.2%	0.8	<0.001	13.1%	2.3%	19.7%	6.9%	1.5	<0.001	21.4%	4.4%	29.4%	9.4%	1.4	<0.001							
PC	44.1%	1.5%	42.5%	2.1%	1.0	<0.01	28.6%	3.0%	34.3%	5.6%	1.2	<0.001	22.8%	2.4%	18.5%	4.7%	0.8	<0.001	2.0%	0.5%	1.3%	0.5%	0.6	<0.001	~3%
PE	18.0%	1.8%	22.5%	4.4%	1.3	<0.001	12.3%	2.8%	17.4%	5.0%	1.4	<0.001	40.1%	2.2%	36.4%	4.2%	0.9	<0.001	28.7%	2.7%	22.6%	5.8%	0.8	<0.001	~1%
PI	46.7%	0.9%	46.7%	2.5%	1.0	n.s.	15.8%	2.9%	21.4%	6.3%	1.4	<0.001	37.5%	2.7%	31.9%	5.5%	0.8	<0.001							
PS	45.6%	0.9%	44.3%	1.6%	1.0	<0.001	30.5%	2.1%	31.2%	3.1%	1.0	n.s.	20.9%	1.8%	20.6%	2.4%	1.0	n.s.							~3%
PG	29.7%	6.6%	23.5%	7.6%	0.8	<0.01	44.7%	2.7%	51.2%	6.2%	1.1	<0.001	22.7%	6.5%	20.0%	7.3%	0.9	<0.05							~4%

*, Approximately 1%-4% chains in PC, PE, PS and PG were not taken into calculation due to absence of its exact compositional information and marked as "missing".
n.s., not significant.

Table S5 Detailed information of related genes and corresponding lipid metabolism pathway obtained by transcriptomics. PCT/ANT, folds change of PCT vs. ANT, calculated as ratio of mean value of PCT vs. ANT. Statistical significance was determined by Wilcoxon Signed-Rank test.

gene	full name	PCT/ANT	*p* value	related lipid pathways
ALOX12B	arachidonate 12-lipoxygenase, 12R type	0.68	0.031	Arachidonic acid metabolism
ALOX15B	arachidonate 15-lipoxygenase, type B	3.85	0.001	Arachidonic acid metabolism
PTGDS	prostaglandin D2 synthase	0.38	0.002	Arachidonic acid metabolism
PTGES3	prostaglandin E synthase 3	1.68	0.000	Arachidonic acid metabolism
PTGIS	prostaglandin I2 synthase	0.45	0.001	Arachidonic acid metabolism
ABCA1	ATP-binding cassette, sub-family A, member 1	1.55	0.007	Cholesterol trafficking
ABCG1	ATP-binding cassette, sub-family G, member 1	1.81	0.002	Cholesterol trafficking
CEPT1	choline/ethanolamine phosphotransferase 1	1.31	0.001	Ether lipid metabolism
EPT1	ethanolaminephosphotransferase 1	1.79	0.000	Ether lipid metabolism
LPCAT1	lysophosphatidylcholine acyltransferase 1	1.62	0.014	Ether lipid metabolism
LPCAT2	lysophosphatidylcholine acyltransferase 2	0.75	0.013	Ether lipid metabolism
PLA2G12A	Phospholipase A2, group XIIA	2.01	0.000	Ether lipid metabolism
PLA2G2A	Phospholipase A2, group IIA	1.36	0.049	Ether lipid metabolism
PLA2G4E	Phospholipase A2, group IVE	2.18	0.018	Ether lipid metabolism
PPAP2A	phosphatidic acid phosphatase type 2A	1.29	0.030	Ether lipid metabolism
ACACA	acetyl-CoA carboxylase alpha	3.24	0.000	Fatty acid biosynthesis
FASN	fatty acid synthase	3.09	0.001	Fatty acid biosynthesis
SCD	stearoyl-CoA desaturase	2.22	0.001	Fatty acid desaturation
ELOVL6	ELOVL fatty acid elongase 6	1.89	0.000	Fatty acid elongation
ELOVL5	ELOVL fatty acid elongase 5	1.80	0.002	Fatty acid elongation
ELOVL2	ELOVL fatty acid elongase 2	2.94	0.000	Fatty acid elongation
ELOVL7	ELOVL fatty acid elongase 7	2.67	0.000	Fatty acid elongation
SREBF1	sterol regulatory element binding transcription factor 1	1.76	0.022	transcriptional regulation of lipogenesis
THRSP	thyroid hormone responsive	2.68	0.008	transcriptional regulation of lipogenesis

LIPE	lipase, hormone-sensitive	0.54	0.007	Glycerolipid metabolism, cholesterol metabolism
PLA2G12A	Phospholipase A2, group XIIA	2.01	0.000	Glycerophospholipid metabolism
LPCAT3	lysophosphatidylcholine acyltransferase 3	1.61	0.000	Glycerophospholipid metabolism
EPT1	ethanolaminephosphotransferase 1	1.79	0.000	Glycerophospholipid metabolism
CEPT1	choline/ethanolamine phosphotransferase 1	1.31	0.001	Glycerophospholipid metabolism
PTDSS1	phosphatidylserine synthase 1	1.61	0.005	Glycerophospholipid metabolism
LPCAT2	lysophosphatidylcholine acyltransferase 2	0.75	0.013	Glycerophospholipid metabolism
LPCAT1	lysophosphatidylcholine acyltransferase 1	1.62	0.014	Glycerophospholipid metabolism
PLA2G4E	Phospholipase A2, group IVE	2.18	0.018	Glycerophospholipid metabolism
PLA2G2A	Phospholipase A2, group IIA	1.36	0.049	Glycerophospholipid metabolism
CRLS1	cardiolipin synthase 1	1.33	0.014	Glycerophospholipid metabolism
CHKA	choline kinase alpha	1.75	0.005	Glycerophospholipid metabolism
PCYT2	phosphate cytidylyltransferase 2, ethanolamine	1.49	0.024	Glycerophospholipid metabolism
CDS1	CDP-diacylglycerol synthase	1.53	0.001	Glycerophospholipid metabolism
MBOAT2	membrane bound O-acyltransferase domain containing 2	3.25	0.000	Glycerophospholipid metabolism, Glycerolipid metabolism
LCLAT1	lysocardiolipin acyltransferase 1	2.56	0.000	Glycerophospholipid metabolism, Glycerolipid metabolism
GK	glycerol kinase	1.38	0.001	Glycerophospholipid metabolism, Glycerolipid metabolism
LPIN2	lipin 2	1.45	0.004	Glycerophospholipid metabolism, Glycerolipid metabolism
DGKG	diacylglycerol kinase, gamma	0.52	0.006	Glycerophospholipid metabolism, Glycerolipid metabolism
DGKD	diacylglycerol kinase, delta	1.68	0.016	Glycerophospholipid metabolism, Glycerolipid metabolism
PPAP2A	phosphatidic acid phosphatase type 2A	1.29	0.030	Glycerophospholipid metabolism, Glycerolipid metabolism
GPAM	glycerol 3-phosphate acyltransferase	1.34	0.030	Glycerophospholipid metabolism, Glycerolipid metabolism
AGPAT3	1-acylglycerol-3-phosphate O-acyltransferase 3	1.65	0.005	Glycerophospholipid metabolism, Glycerolipid metabolism
SLC27A2	solute carrier family 27 (fatty acid transporter), member 2	1.89	0.001	lipid uptaking/transporting
SLC27A4	solute carrier family 27 (fatty acid transporter), member 4	1.55	0.022	lipid uptaking/transporting
SLC27A5	solute carrier family 27 (fatty acid transporter), member 5	1.59	0.009	lipid uptaking/transporting
SCARB1	scavenger receptor class B, member 1	1.47	0.011	lipid uptaking/transporting

GOT2	glutamic-oxaloacetic transaminase 2, mitochondrial	1.47	0.011	lipid uptaking/transporting
ACER1	alkaline ceramidase 1	0.37	0.001	Sphingolipid metabolism
ACER2	alkaline ceramidase 2	1.70	0.002	Sphingolipid metabolism
ASAH1	N-acylsphingosine amidohydrolase (acid ceramidase) 1	1.87	0.001	Sphingolipid metabolism
ASAH2	N-acylsphingosine amidohydrolase (acid ceramidase) 2	1.36	0.032	Sphingolipid metabolism
CERS1	ceramide synthase 1	2.48	0.004	Sphingolipid metabolism
CERS2	ceramide synthase 2	1.61	0.001	Sphingolipid metabolism
CERS3	ceramide synthase 3	0.46	0.024	Sphingolipid metabolism
CERS4	ceramide synthase 4	2.74	0.000	Sphingolipid metabolism
CERS6	ceramide synthase 6	1.52	0.003	Sphingolipid metabolism
DEGS1	delta(4)-desaturase, sphingolipid 1	1.76	0.001	Sphingolipid metabolism
PPAP2A	phosphatidic acid phosphatase type 2A	1.29	0.030	Sphingolipid metabolism
SGMS1	sphingomyelin synthase 1	1.73	0.000	Sphingolipid metabolism
SGMS2	sphingomyelin synthase 2	1.27	0.008	Sphingolipid metabolism
SGPP1	sphingosine-1-phosphate phosphatase 1	1.90	0.000	Sphingolipid metabolism
SGPP2	sphingosine-1-phosphate phosphatase 2	1.69	0.019	Sphingolipid metabolism
SMPD2	neutral sphingomyelinase	1.77	0.043	Sphingolipid metabolism
SPTLC2	serine palmitoyltransferase, long chain base subunit 2	1.52	0.001	Sphingolipid metabolism
FDPS	farnesyl diphosphate synthase	1.48	0.018	Steroid biosynthesis
FDFT1	farnesyl-diphosphate farnesyltransferase 1	1.38	0.012	Steroid biosynthesis
SQLE	squalene epoxidase	1.31	0.046	Steroid biosynthesis
SOAT1	sterol O-acyltransferase 1	1.43	0.011	Steroid biosynthesis

Table S6 Detailed information of related miRNAs, their target genes and corresponding lipid metabolism pathway obtained by transcriptomics. PCT/ANT, folds change of PCT vs. ANT, calculated as ratio of mean value of PCT vs. ANT. Statistical significance was determined by Wilcoxon Signed-Rank test.

miRNA	target gene	PCT/ANT	*p* value	related lipid pathway
hsa-miR-222-3p	ACACA	0.51	0.000	Fatty acid biosynthesis
hsa-miR-24-3p	ACACA	0.75	0.021	Fatty acid biosynthesis
hsa-miR-222-3p	FASN	0.51	0.000	Fatty acid biosynthesis
hsa-miR-26a-5p	FASN	0.86	0.007	Fatty acid biosynthesis
hsa-miR-27b-3p	FASN	0.72	0.005	Fatty acid biosynthesis
hsa-miR-331-3p	FASN	0.42	0.012	Fatty acid biosynthesis
hsa-miR-92b-3p	FASN	0.74	0.002	Fatty acid biosynthesis
hsa-let-7e-5p	SCD	0.73	0.001	Fatty acid desaturation
hsa-miR-331-3p	SCD	0.42	0.012	Fatty acid desaturation
hsa-miR-455-3p	SCD	0.52	0.001	Fatty acid desaturation
hsa-miR-124-3p	ELOVL5	0.76	0.012	Fatty acid elongation
hsa-miR-204-5p	ELOVL6	0.58	0.002	Fatty acid elongation
hsa-miR-26a-5p	ELOVL6	0.86	0.007	Fatty acid elongation
hsa-miR-124-3p	CEPT1	0.76	0.012	Glycerophospholipid metabolism, Ether lipid metabolism
hsa-miR-23b-3p	CEPT1	0.77	0.034	Glycerophospholipid metabolism, Ether lipid metabolism
hsa-miR-26a-5p	CEPT1	0.86	0.007	Glycerophospholipid metabolism, Ether lipid metabolism
hsa-miR-92b-3p	PLA2G12A	0.74	0.002	Glycerophospholipid metabolism, Ether lipid metabolism
hsa-miR-24-3p	AGPAT3	0.75	0.021	Glycerophospholipid metabolism, Glycerolipid metabolism
hsa-miR-100-5p	AGPAT3	0.73	0.002	Glycerophospholipid metabolism, Glycerolipid metabolism
hsa-miR-27a-3p	GPAM	0.73	0.007	Glycerophospholipid metabolism, Glycerolipid metabolism
hsa-miR-124-3p	LCLAT1	0.76	0.012	Glycerophospholipid metabolism, Glycerolipid metabolism
hsa-miR-125a-5p	SCARB1	0.71	0.000	lipid uptaking/transporting
hsa-miR-455-3p	SCARB1	0.52	0.001	lipid uptaking/transporting
hsa-miR-455-5p	SCARB1	0.55	0.000	lipid uptaking/transporting
hsa-miR-1	SLC27A4	0.56	0.001	lipid uptaking/transporting
hsa-miR-1	CERS2	0.56	0.001	Sphingolipid metabolism
hsa-miR-124-3p	CERS2	0.76	0.012	Sphingolipid metabolism
hsa-miR-221-3p	CERS2	0.52	0.001	Sphingolipid metabolism
hsa-miR-222-3p	CERS2	0.51	0.000	Sphingolipid metabolism
hsa-miR-124-3p	SGMS1	0.76	0.012	Sphingolipid metabolism
hsa-let-7e-5p	FDPS	0.73	0.001	Steroid biosynthesis
hsa-let-7e-5p	SQLE	0.73	0.001	Steroid biosynthesis

Table S7 Detailed statistical information of 9 most prominently elevated lipid species selected according to *p* value, AUC and PCT-to-ANT folds change, i.e., scheme shown in Fig. 3A

	PCT vs. ANT				95% Confidence Interval			
lipids	*p* value	PCT/ANT	AUC	Std. Error	Lower	Upper	Sensitivity	Specificity
CE 24:5	<0.001	35.46	0.94	0.03	0.88	1.00	0.96	0.80
CE 24:4	<0.001	24.86	0.94	0.03	0.87	1.00	0.96	0.88
CE 20:1	<0.001	45.48	0.94	0.03	0.87	1.00	0.72	1.00
CE 18:1	<0.001	11.78	0.93	0.04	0.86	1.00	0.84	0.92
Cer(d18:1/22:1)	<0.001	5.99	0.91	0.05	0.82	1.00	0.88	0.92
FFA22:3	<0.001	5.39	0.91	0.04	0.83	0.99	0.96	0.72
CE 22:6	<0.001	10.48	0.90	0.04	0.82	0.98	0.84	0.80
TAG 58:1	<0.001	6.25	0.89	0.05	0.80	0.98	0.88	0.84
Cer(d18:2/20:0)	<0.001	11.38	0.89	0.05	0.78	0.99	0.80	1.00

Table S8 Differential lipids significantly associated with PCa progression assessed by Gleason Score grades (a) and metastatic grades, i.e., localized, locally advanced and metastatic grade (b).

Table S8a

	PCT vs ANT		PCT-to-ANT[a] ratio in GS grades			
lipid	FC[a]	*p* value[b]	GS<7 (n=2)	GS=7 (n=12)	GS>7 (n=11)	*p* value[c]
CE 22:6	10.48	<0.001	0.5	4.6	23.6	0.006
CE 24:4	24.86	<0.001	0.8	22.9	48.5	0.014
FFA20:2	3.90	<0.001	0.8	2.9	6.9	0.040
FFA20:4	2.61	<0.001	0.7	2.3	3.6	0.039
FFA20:5	2.49	0.002	0.7	1.7	4.1	0.033
FFA22:2	4.43	<0.001	0.7	3.2	8.6	0.026
FFA22:3	5.39	<0.001	1.0	4.2	9.7	0.026
FFA22:5	3.06	0.001	0.7	2.7	4.1	0.039
PG 36:3	2.87	0.002	0.5	2.1	5.8	0.031

Table S8b

	PCT vs ANT		PCT-to-ANT[a] ratio in metastatic grades		
lipid	FC[a]	*p* value[b]	localized(n=16)	locally advanced and metastatic (n=7)	*p* value[d]
CE 22:6	10.5	<0.001	5.5	46.7	0.033
CE 24:4	24.9	<0.001	14.5	149.9	0.038
CE 24:5	35.5	<0.001	14.2	259.7	0.016
TAG 54:1	3.5	0.001	2.0	25.4	0.038
TAG 55:2	3.2	0.003	1.6	47.9	0.019
TAG 56:2	3.5	0.002	1.8	28.9	0.038
TAG 58:4	2.6	0.005	1.0	19.5	0.033
TAG 58:5	2.7	0.009	1.0	20.9	0.033
TAG 60:5	4.5	0.002	1.7	66.6	0.013
TAG 60:6	5.2	0.004	1.8	46.1	0.013

a, FC(folds change) and PCT-to-ANT ratio are calculated as ratio of mean value of PCT vs. ANT;

b, statistical significance determined by Wilcoxon Signed-Rank test;

c, statistical significance determined by Kruskal-Wallis test;

d, statistical significance determined by Mann-Whitney test

References

1. Wallner, S. & Schmitz, G. Plasmalogens the neglected regulatory and scavenging lipid species. *Chem Phys Lipids* **164**, 573-589 (2011).
2. Paschos, A., Pandya, R., Duivenvoorden, W. C. M. & Pinthus, J. H. Oxidative stress in prostate cancer: changing research concepts towards a novel paradigm for prevention and therapeutics. *Prostate Cancer P D* **16**, 217-225 (2013).
